# Multi-channel search for squarks and gluinos in $\sqrt{s}=7\mbox{ TeV}$*pp* collisions with the ATLAS detector at the LHC

**DOI:** 10.1140/epjc/s10052-013-2362-5

**Published:** 2013-03-27

**Authors:** The ATLAS Collaboration, G. Aad, T. Abajyan, B. Abbott, J. Abdallah, S. Abdel Khalek, A. A. Abdelalim, O. Abdinov, R. Aben, B. Abi, M. Abolins, O. S. AbouZeid, H. Abramowicz, H. Abreu, B. S. Acharya, L. Adamczyk, D. L. Adams, T. N. Addy, J. Adelman, S. Adomeit, P. Adragna, T. Adye, S. Aefsky, J. A. Aguilar-Saavedra, M. Agustoni, S. P. Ahlen, F. Ahles, A. Ahmad, M. Ahsan, G. Aielli, T. P. A. Åkesson, G. Akimoto, A. V. Akimov, M. A. Alam, J. Albert, S. Albrand, M. Aleksa, I. N. Aleksandrov, F. Alessandria, C. Alexa, G. Alexander, G. Alexandre, T. Alexopoulos, M. Alhroob, M. Aliev, G. Alimonti, J. Alison, B. M. M. Allbrooke, L. J. Allison, P. P. Allport, S. E. Allwood-Spiers, J. Almond, A. Aloisio, R. Alon, A. Alonso, F. Alonso, A. Altheimer, B. Alvarez Gonzalez, M. G. Alviggi, K. Amako, C. Amelung, V. V. Ammosov, S. P. Amor Dos Santos, A. Amorim, S. Amoroso, N. Amram, C. Anastopoulos, L. S. Ancu, N. Andari, T. Andeen, C. F. Anders, G. Anders, K. J. Anderson, A. Andreazza, V. Andrei, M-L. Andrieux, X. S. Anduaga, S. Angelidakis, P. Anger, A. Angerami, F. Anghinolfi, A. Anisenkov, N. Anjos, A. Annovi, A. Antonaki, M. Antonelli, A. Antonov, J. Antos, F. Anulli, M. Aoki, S. Aoun, L. Aperio Bella, R. Apolle, G. Arabidze, I. Aracena, Y. Arai, A. T. H. Arce, S. Arfaoui, J-F. Arguin, S. Argyropoulos, E. Arik, M. Arik, A. J. Armbruster, O. Arnaez, V. Arnal, A. Artamonov, G. Artoni, D. Arutinov, S. Asai, S. Ask, B. Åsman, L. Asquith, K. Assamagan, A. Astbury, M. Atkinson, B. Aubert, E. Auge, K. Augsten, M. Aurousseau, G. Avolio, D. Axen, G. Azuelos, Y. Azuma, M. A. Baak, G. Baccaglioni, C. Bacci, A. M. Bach, H. Bachacou, K. Bachas, M. Backes, M. Backhaus, J. Backus Mayes, E. Badescu, P. Bagnaia, Y. Bai, D. C. Bailey, T. Bain, J. T. Baines, O. K. Baker, S. Baker, P. Balek, E. Banas, P. Banerjee, Sw. Banerjee, D. Banfi, A. Bangert, V. Bansal, H. S. Bansil, L. Barak, S. P. Baranov, T. Barber, E. L. Barberio, D. Barberis, M. Barbero, D. Y. Bardin, T. Barillari, M. Barisonzi, T. Barklow, N. Barlow, B. M. Barnett, R. M. Barnett, A. Baroncelli, G. Barone, A. J. Barr, F. Barreiro, J. Barreiro Guimarães da Costa, R. Bartoldus, A. E. Barton, V. Bartsch, A. Basye, R. L. Bates, L. Batkova, J. R. Batley, A. Battaglia, M. Battistin, F. Bauer, H. S. Bawa, S. Beale, T. Beau, P. H. Beauchemin, R. Beccherle, P. Bechtle, H. P. Beck, K. Becker, S. Becker, M. Beckingham, K. H. Becks, A. J. Beddall, A. Beddall, S. Bedikian, V. A. Bednyakov, C. P. Bee, L. J. Beemster, M. Begel, S. Behar Harpaz, P. K. Behera, M. Beimforde, C. Belanger-Champagne, P. J. Bell, W. H. Bell, G. Bella, L. Bellagamba, M. Bellomo, A. Belloni, O. Beloborodova, K. Belotskiy, O. Beltramello, O. Benary, D. Benchekroun, K. Bendtz, N. Benekos, Y. Benhammou, E. Benhar Noccioli, J. A. Benitez Garcia, D. P. Benjamin, M. Benoit, J. R. Bensinger, K. Benslama, S. Bentvelsen, D. Berge, E. Bergeaas Kuutmann, N. Berger, F. Berghaus, E. Berglund, J. Beringer, P. Bernat, R. Bernhard, C. Bernius, T. Berry, C. Bertella, A. Bertin, F. Bertolucci, M. I. Besana, G. J. Besjes, N. Besson, S. Bethke, W. Bhimji, R. M. Bianchi, L. Bianchini, M. Bianco, O. Biebel, S. P. Bieniek, K. Bierwagen, J. Biesiada, M. Biglietti, H. Bilokon, M. Bindi, S. Binet, A. Bingul, C. Bini, C. Biscarat, B. Bittner, C. W. Black, K. M. Black, R. E. Blair, J.-B. Blanchard, T. Blazek, I. Bloch, C. Blocker, J. Blocki, W. Blum, U. Blumenschein, G. J. Bobbink, V. S. Bobrovnikov, S. S. Bocchetta, A. Bocci, C. R. Boddy, M. Boehler, J. Boek, T. T. Boek, N. Boelaert, J. A. Bogaerts, A. Bogdanchikov, A. Bogouch, C. Bohm, J. Bohm, V. Boisvert, T. Bold, V. Boldea, N. M. Bolnet, M. Bomben, M. Bona, M. Boonekamp, S. Bordoni, C. Borer, A. Borisov, G. Borissov, I. Borjanovic, M. Borri, S. Borroni, J. Bortfeldt, V. Bortolotto, K. Bos, D. Boscherini, M. Bosman, H. Boterenbrood, J. Bouchami, J. Boudreau, E. V. Bouhova-Thacker, D. Boumediene, C. Bourdarios, N. Bousson, A. Boveia, J. Boyd, I. R. Boyko, I. Bozovic-Jelisavcic, J. Bracinik, P. Branchini, A. Brandt, G. Brandt, O. Brandt, U. Bratzler, B. Brau, J. E. Brau, H. M. Braun, S. F. Brazzale, B. Brelier, J. Bremer, K. Brendlinger, R. Brenner, S. Bressler, T. M. Bristow, D. Britton, F. M. Brochu, I. Brock, R. Brock, F. Broggi, C. Bromberg, J. Bronner, G. Brooijmans, T. Brooks, W. K. Brooks, G. Brown, P. A. Bruckman de Renstrom, D. Bruncko, R. Bruneliere, S. Brunet, A. Bruni, G. Bruni, M. Bruschi, L. Bryngemark, T. Buanes, Q. Buat, F. Bucci, J. Buchanan, P. Buchholz, R. M. Buckingham, A. G. Buckley, S. I. Buda, I. A. Budagov, B. Budick, V. Büscher, L. Bugge, O. Bulekov, A. C. Bundock, M. Bunse, T. Buran, H. Burckhart, S. Burdin, T. Burgess, S. Burke, E. Busato, P. Bussey, C. P. Buszello, B. Butler, J. M. Butler, C. M. Buttar, J. M. Butterworth, W. Buttinger, M. Byszewski, S. Cabrera Urbán, D. Caforio, O. Cakir, P. Calafiura, G. Calderini, P. Calfayan, R. Calkins, L. P. Caloba, R. Caloi, D. Calvet, S. Calvet, R. Camacho Toro, P. Camarri, D. Cameron, L. M. Caminada, R. Caminal Armadans, S. Campana, M. Campanelli, V. Canale, F. Canelli, A. Canepa, J. Cantero, R. Cantrill, M. D. M. Capeans Garrido, I. Caprini, M. Caprini, D. Capriotti, M. Capua, R. Caputo, R. Cardarelli, T. Carli, G. Carlino, L. Carminati, S. Caron, E. Carquin, G. D. Carrillo-Montoya, A. A. Carter, J. R. Carter, J. Carvalho, D. Casadei, M. P. Casado, M. Cascella, C. Caso, A. M. Castaneda Hernandez, E. Castaneda-Miranda, V. Castillo Gimenez, N. F. Castro, G. Cataldi, P. Catastini, A. Catinaccio, J. R. Catmore, A. Cattai, G. Cattani, S. Caughron, V. Cavaliere, P. Cavalleri, D. Cavalli, M. Cavalli-Sforza, V. Cavasinni, F. Ceradini, A. S. Cerqueira, A. Cerri, L. Cerrito, F. Cerutti, S. A. Cetin, A. Chafaq, D. Chakraborty, I. Chalupkova, K. Chan, P. Chang, B. Chapleau, J. D. Chapman, J. W. Chapman, D. G. Charlton, V. Chavda, C. A. Chavez Barajas, S. Cheatham, S. Chekanov, S. V. Chekulaev, G. A. Chelkov, M. A. Chelstowska, C. Chen, H. Chen, S. Chen, X. Chen, Y. Chen, Y. Cheng, A. Cheplakov, R. Cherkaoui El Moursli, V. Chernyatin, E. Cheu, S. L. Cheung, L. Chevalier, G. Chiefari, L. Chikovani, J. T. Childers, A. Chilingarov, G. Chiodini, A. S. Chisholm, R. T. Chislett, A. Chitan, M. V. Chizhov, G. Choudalakis, S. Chouridou, I. A. Christidi, A. Christov, D. Chromek-Burckhart, M. L. Chu, J. Chudoba, G. Ciapetti, A. K. Ciftci, R. Ciftci, D. Cinca, V. Cindro, A. Ciocio, M. Cirilli, P. Cirkovic, Z. H. Citron, M. Citterio, M. Ciubancan, A. Clark, P. J. Clark, R. N. Clarke, W. Cleland, J. C. Clemens, B. Clement, C. Clement, Y. Coadou, M. Cobal, A. Coccaro, J. Cochran, L. Coffey, J. G. Cogan, J. Coggeshall, J. Colas, S. Cole, A. P. Colijn, N. J. Collins, C. Collins-Tooth, J. Collot, T. Colombo, G. Colon, G. Compostella, P. Conde Muiño, E. Coniavitis, M. C. Conidi, S. M. Consonni, V. Consorti, S. Constantinescu, C. Conta, G. Conti, F. Conventi, M. Cooke, B. D. Cooper, A. M. Cooper-Sarkar, K. Copic, T. Cornelissen, M. Corradi, F. Corriveau, A. Cortes-Gonzalez, G. Cortiana, G. Costa, M. J. Costa, D. Costanzo, D. Côté, L. Courneyea, G. Cowan, B. E. Cox, K. Cranmer, F. Crescioli, M. Cristinziani, G. Crosetti, S. Crépé-Renaudin, C.-M. Cuciuc, C. Cuenca Almenar, T. Cuhadar Donszelmann, J. Cummings, M. Curatolo, C. J. Curtis, C. Cuthbert, P. Cwetanski, H. Czirr, P. Czodrowski, Z. Czyczula, S. D’Auria, M. D’Onofrio, A. D’Orazio, M. J. Da Cunha Sargedas De Sousa, C. Da Via, W. Dabrowski, A. Dafinca, T. Dai, F. Dallaire, C. Dallapiccola, M. Dam, M. Dameri, D. S. Damiani, H. O. Danielsson, V. Dao, G. Darbo, G. L. Darlea, J. A. Dassoulas, W. Davey, T. Davidek, N. Davidson, R. Davidson, E. Davies, M. Davies, O. Davignon, A. R. Davison, Y. Davygora, E. Dawe, I. Dawson, R. K. Daya-Ishmukhametova, K. De, R. de Asmundis, S. De Castro, S. De Cecco, J. de Graat, N. De Groot, P. de Jong, C. De La Taille, H. De la Torre, F. De Lorenzi, L. De Nooij, D. De Pedis, A. De Salvo, U. De Sanctis, A. De Santo, J. B. De Vivie De Regie, G. De Zorzi, W. J. Dearnaley, R. Debbe, C. Debenedetti, B. Dechenaux, D. V. Dedovich, J. Degenhardt, J. Del Peso, T. Del Prete, T. Delemontex, M. Deliyergiyev, A. Dell’Acqua, L. Dell’Asta, M. Della Pietra, D. della Volpe, M. Delmastro, P. A. Delsart, C. Deluca, S. Demers, M. Demichev, B. Demirkoz, S. P. Denisov, D. Derendarz, J. E. Derkaoui, F. Derue, P. Dervan, K. Desch, E. Devetak, P. O. Deviveiros, A. Dewhurst, B. DeWilde, S. Dhaliwal, R. Dhullipudi, A. Di Ciaccio, L. Di Ciaccio, C. Di Donato, A. Di Girolamo, B. Di Girolamo, S. Di Luise, A. Di Mattia, B. Di Micco, R. Di Nardo, A. Di Simone, R. Di Sipio, M. A. Diaz, E. B. Diehl, J. Dietrich, T. A. Dietzsch, S. Diglio, K. Dindar Yagci, J. Dingfelder, F. Dinut, C. Dionisi, P. Dita, S. Dita, F. Dittus, F. Djama, T. Djobava, M. A. B. do Vale, A. Do Valle Wemans, T. K. O. Doan, M. Dobbs, D. Dobos, E. Dobson, J. Dodd, C. Doglioni, T. Doherty, Y. Doi, J. Dolejsi, Z. Dolezal, B. A. Dolgoshein, T. Dohmae, M. Donadelli, J. Donini, J. Dopke, A. Doria, A. Dos Anjos, A. Dotti, M. T. Dova, A. D. Doxiadis, A. T. Doyle, N. Dressnandt, M. Dris, J. Dubbert, S. Dube, E. Dubreuil, E. Duchovni, G. Duckeck, D. Duda, A. Dudarev, F. Dudziak, M. Dührssen, I. P. Duerdoth, L. Duflot, M-A. Dufour, L. Duguid, M. Dunford, H. Duran Yildiz, R. Duxfield, M. Dwuznik, M. Düren, W. L. Ebenstein, J. Ebke, S. Eckweiler, W. Edson, C. A. Edwards, N. C. Edwards, W. Ehrenfeld, T. Eifert, G. Eigen, K. Einsweiler, E. Eisenhandler, T. Ekelof, M. El Kacimi, M. Ellert, S. Elles, F. Ellinghaus, K. Ellis, N. Ellis, J. Elmsheuser, M. Elsing, D. Emeliyanov, R. Engelmann, A. Engl, B. Epp, J. Erdmann, A. Ereditato, D. Eriksson, J. Ernst, M. Ernst, J. Ernwein, D. Errede, S. Errede, E. Ertel, M. Escalier, H. Esch, C. Escobar, X. Espinal Curull, B. Esposito, F. Etienne, A. I. Etienvre, E. Etzion, D. Evangelakou, H. Evans, L. Fabbri, C. Fabre, R. M. Fakhrutdinov, S. Falciano, Y. Fang, M. Fanti, A. Farbin, A. Farilla, J. Farley, T. Farooque, S. Farrell, S. M. Farrington, P. Farthouat, F. Fassi, P. Fassnacht, D. Fassouliotis, B. Fatholahzadeh, A. Favareto, L. Fayard, P. Federic, O. L. Fedin, W. Fedorko, M. Fehling-Kaschek, L. Feligioni, C. Feng, E. J. Feng, A. B. Fenyuk, J. Ferencei, W. Fernando, S. Ferrag, J. Ferrando, V. Ferrara, A. Ferrari, P. Ferrari, R. Ferrari, D. E. Ferreira de Lima, A. Ferrer, D. Ferrere, C. Ferretti, A. Ferretto Parodi, M. Fiascaris, F. Fiedler, A. Filipčič, F. Filthaut, M. Fincke-Keeler, M. C. N. Fiolhais, L. Fiorini, A. Firan, G. Fischer, M. J. Fisher, E. A. Fitzgerald, M. Flechl, I. Fleck, J. Fleckner, P. Fleischmann, S. Fleischmann, G. Fletcher, T. Flick, A. Floderus, L. R. Flores Castillo, A. C. Florez Bustos, M. J. Flowerdew, T. Fonseca Martin, A. Formica, A. Forti, D. Fortin, D. Fournier, A. J. Fowler, H. Fox, P. Francavilla, M. Franchini, S. Franchino, D. Francis, T. Frank, M. Franklin, S. Franz, M. Fraternali, S. Fratina, S. T. French, C. Friedrich, F. Friedrich, D. Froidevaux, J. A. Frost, C. Fukunaga, E. Fullana Torregrosa, B. G. Fulsom, J. Fuster, C. Gabaldon, O. Gabizon, S. Gadatsch, T. Gadfort, S. Gadomski, G. Gagliardi, P. Gagnon, C. Galea, B. Galhardo, E. J. Gallas, V. Gallo, B. J. Gallop, P. Gallus, K. K. Gan, Y. S. Gao, A. Gaponenko, F. Garberson, M. Garcia-Sciveres, C. García, J. E. García Navarro, R. W. Gardner, N. Garelli, V. Garonne, C. Gatti, G. Gaudio, B. Gaur, L. Gauthier, P. Gauzzi, I. L. Gavrilenko, C. Gay, G. Gaycken, E. N. Gazis, P. Ge, Z. Gecse, C. N. P. Gee, D. A. A. Geerts, Ch. Geich-Gimbel, K. Gellerstedt, C. Gemme, A. Gemmell, M. H. Genest, S. Gentile, M. George, S. George, D. Gerbaudo, P. Gerlach, A. Gershon, C. Geweniger, H. Ghazlane, N. Ghodbane, B. Giacobbe, S. Giagu, V. Giangiobbe, F. Gianotti, B. Gibbard, A. Gibson, S. M. Gibson, M. Gilchriese, T. P. S. Gillam, D. Gillberg, A. R. Gillman, D. M. Gingrich, J. Ginzburg, N. Giokaris, M. P. Giordani, R. Giordano, F. M. Giorgi, P. Giovannini, P. F. Giraud, D. Giugni, M. Giunta, B. K. Gjelsten, L. K. Gladilin, C. Glasman, J. Glatzer, A. Glazov, G. L. Glonti, J. R. Goddard, J. Godfrey, J. Godlewski, M. Goebel, T. Göpfert, C. Goeringer, C. Gössling, S. Goldfarb, T. Golling, D. Golubkov, A. Gomes, L. S. Gomez Fajardo, R. Gonçalo, J. Goncalves Pinto Firmino Da Costa, L. Gonella, S. González de la Hoz, G. Gonzalez Parra, M. L. Gonzalez Silva, S. Gonzalez-Sevilla, J. J. Goodson, L. Goossens, P. A. Gorbounov, H. A. Gordon, I. Gorelov, G. Gorfine, B. Gorini, E. Gorini, A. Gorišek, E. Gornicki, A. T. Goshaw, M. Gosselink, M. I. Gostkin, I. Gough Eschrich, M. Gouighri, D. Goujdami, M. P. Goulette, A. G. Goussiou, C. Goy, S. Gozpinar, I. Grabowska-Bold, P. Grafström, K-J. Grahn, E. Gramstad, F. Grancagnolo, S. Grancagnolo, V. Grassi, V. Gratchev, H. M. Gray, J. A. Gray, E. Graziani, O. G. Grebenyuk, T. Greenshaw, Z. D. Greenwood, K. Gregersen, I. M. Gregor, P. Grenier, J. Griffiths, N. Grigalashvili, A. A. Grillo, K. Grimm, S. Grinstein, Ph. Gris, Y. V. Grishkevich, J.-F. Grivaz, A. Grohsjean, E. Gross, J. Grosse-Knetter, J. Groth-Jensen, K. Grybel, D. Guest, C. Guicheney, E. Guido, T. Guillemin, S. Guindon, U. Gul, J. Gunther, B. Guo, J. Guo, P. Gutierrez, N. Guttman, O. Gutzwiller, C. Guyot, C. Gwenlan, C. B. Gwilliam, A. Haas, S. Haas, C. Haber, H. K. Hadavand, D. R. Hadley, P. Haefner, F. Hahn, Z. Hajduk, H. Hakobyan, D. Hall, G. Halladjian, K. Hamacher, P. Hamal, K. Hamano, M. Hamer, A. Hamilton, S. Hamilton, L. Han, K. Hanagaki, K. Hanawa, M. Hance, C. Handel, P. Hanke, J. R. Hansen, J. B. Hansen, J. D. Hansen, P. H. Hansen, P. Hansson, K. Hara, T. Harenberg, S. Harkusha, D. Harper, R. D. Harrington, O. M. Harris, J. Hartert, F. Hartjes, T. Haruyama, A. Harvey, S. Hasegawa, Y. Hasegawa, S. Hassani, S. Haug, M. Hauschild, R. Hauser, M. Havranek, C. M. Hawkes, R. J. Hawkings, A. D. Hawkins, T. Hayakawa, T. Hayashi, D. Hayden, C. P. Hays, H. S. Hayward, S. J. Haywood, S. J. Head, V. Hedberg, L. Heelan, S. Heim, B. Heinemann, S. Heisterkamp, L. Helary, C. Heller, M. Heller, S. Hellman, D. Hellmich, C. Helsens, R. C. W. Henderson, M. Henke, A. Henrichs, A. M. Henriques Correia, S. Henrot-Versille, C. Hensel, C. M. Hernandez, Y. Hernández Jiménez, R. Herrberg, G. Herten, R. Hertenberger, L. Hervas, G. G. Hesketh, N. P. Hessey, R. Hickling, E. Higón-Rodriguez, J. C. Hill, K. H. Hiller, S. Hillert, S. J. Hillier, I. Hinchliffe, E. Hines, M. Hirose, F. Hirsch, D. Hirschbuehl, J. Hobbs, N. Hod, M. C. Hodgkinson, P. Hodgson, A. Hoecker, M. R. Hoeferkamp, J. Hoffman, D. Hoffmann, M. Hohlfeld, M. Holder, S. O. Holmgren, T. Holy, J. L. Holzbauer, T. M. Hong, L. Hooft van Huysduynen, S. Horner, J-Y. Hostachy, S. Hou, A. Hoummada, J. Howard, J. Howarth, M. Hrabovsky, I. Hristova, J. Hrivnac, T. Hryn’ova, P. J. Hsu, S.-C. Hsu, D. Hu, Z. Hubacek, F. Hubaut, F. Huegging, A. Huettmann, T. B. Huffman, E. W. Hughes, G. Hughes, M. Huhtinen, M. Hurwitz, N. Huseynov, J. Huston, J. Huth, G. Iacobucci, G. Iakovidis, M. Ibbotson, I. Ibragimov, L. Iconomidou-Fayard, J. Idarraga, P. Iengo, O. Igonkina, Y. Ikegami, M. Ikeno, D. Iliadis, N. Ilic, T. Ince, P. Ioannou, M. Iodice, K. Iordanidou, V. Ippolito, A. Irles Quiles, C. Isaksson, M. Ishino, M. Ishitsuka, R. Ishmukhametov, C. Issever, S. Istin, A. V. Ivashin, W. Iwanski, H. Iwasaki, J. M. Izen, V. Izzo, B. Jackson, J. N. Jackson, P. Jackson, M. R. Jaekel, V. Jain, K. Jakobs, S. Jakobsen, T. Jakoubek, J. Jakubek, D. O. Jamin, D. K. Jana, E. Jansen, H. Jansen, J. Janssen, A. Jantsch, M. Janus, R. C. Jared, G. Jarlskog, L. Jeanty, I. Jen-La Plante, G.-Y. Jeng, D. Jennens, P. Jenni, A. E. Loevschall-Jensen, P. Jež, S. Jézéquel, M. K. Jha, H. Ji, W. Ji, J. Jia, Y. Jiang, M. Jimenez Belenguer, S. Jin, O. Jinnouchi, M. D. Joergensen, D. Joffe, M. Johansen, K. E. Johansson, P. Johansson, S. Johnert, K. A. Johns, K. Jon-And, G. Jones, R. W. L. Jones, T. J. Jones, C. Joram, P. M. Jorge, K. D. Joshi, J. Jovicevic, T. Jovin, X. Ju, C. A. Jung, R. M. Jungst, V. Juranek, P. Jussel, A. Juste Rozas, S. Kabana, M. Kaci, A. Kaczmarska, P. Kadlecik, M. Kado, H. Kagan, M. Kagan, E. Kajomovitz, S. Kalinin, L. V. Kalinovskaya, S. Kama, N. Kanaya, M. Kaneda, S. Kaneti, T. Kanno, V. A. Kantserov, J. Kanzaki, B. Kaplan, A. Kapliy, D. Kar, M. Karagounis, K. Karakostas, M. Karnevskiy, V. Kartvelishvili, A. N. Karyukhin, L. Kashif, G. Kasieczka, R. D. Kass, A. Kastanas, Y. Kataoka, J. Katzy, V. Kaushik, K. Kawagoe, T. Kawamoto, G. Kawamura, S. Kazama, V. F. Kazanin, M. Y. Kazarinov, R. Keeler, P. T. Keener, R. Kehoe, M. Keil, G. D. Kekelidze, J. S. Keller, M. Kenyon, H. Keoshkerian, O. Kepka, N. Kerschen, B. P. Kerševan, S. Kersten, K. Kessoku, J. Keung, F. Khalil-zada, H. Khandanyan, A. Khanov, D. Kharchenko, A. Khodinov, A. Khomich, T. J. Khoo, G. Khoriauli, A. Khoroshilov, V. Khovanskiy, E. Khramov, J. Khubua, H. Kim, S. H. Kim, N. Kimura, O. Kind, B. T. King, M. King, R. S. B. King, J. Kirk, A. E. Kiryunin, T. Kishimoto, D. Kisielewska, T. Kitamura, T. Kittelmann, K. Kiuchi, E. Kladiva, M. Klein, U. Klein, K. Kleinknecht, M. Klemetti, A. Klier, P. Klimek, A. Klimentov, R. Klingenberg, J. A. Klinger, E. B. Klinkby, T. Klioutchnikova, P. F. Klok, S. Klous, E.-E. Kluge, T. Kluge, P. Kluit, S. Kluth, E. Kneringer, E. B. F. G. Knoops, A. Knue, B. R. Ko, T. Kobayashi, M. Kobel, M. Kocian, P. Kodys, K. Köneke, A. C. König, S. Koenig, L. Köpke, F. Koetsveld, P. Koevesarki, T. Koffas, E. Koffeman, L. A. Kogan, S. Kohlmann, F. Kohn, Z. Kohout, T. Kohriki, T. Koi, G. M. Kolachev, H. Kolanoski, V. Kolesnikov, I. Koletsou, J. Koll, A. A. Komar, Y. Komori, T. Kondo, T. Kono, A. I. Kononov, R. Konoplich, N. Konstantinidis, R. Kopeliansky, S. Koperny, A. K. Kopp, K. Korcyl, K. Kordas, A. Korn, A. Korol, I. Korolkov, E. V. Korolkova, V. A. Korotkov, O. Kortner, S. Kortner, V. V. Kostyukhin, S. Kotov, V. M. Kotov, A. Kotwal, C. Kourkoumelis, V. Kouskoura, A. Koutsman, R. Kowalewski, T. Z. Kowalski, W. Kozanecki, A. S. Kozhin, V. Kral, V. A. Kramarenko, G. Kramberger, M. W. Krasny, A. Krasznahorkay, J. K. Kraus, A. Kravchenko, S. Kreiss, F. Krejci, J. Kretzschmar, K. Kreutzfeldt, N. Krieger, P. Krieger, K. Kroeninger, H. Kroha, J. Kroll, J. Kroseberg, J. Krstic, U. Kruchonak, H. Krüger, T. Kruker, N. Krumnack, Z. V. Krumshteyn, M. K. Kruse, T. Kubota, S. Kuday, S. Kuehn, A. Kugel, T. Kuhl, V. Kukhtin, Y. Kulchitsky, S. Kuleshov, M. Kuna, J. Kunkle, A. Kupco, H. Kurashige, M. Kurata, Y. A. Kurochkin, V. Kus, E. S. Kuwertz, M. Kuze, J. Kvita, R. Kwee, A. La Rosa, L. La Rotonda, L. Labarga, S. Lablak, C. Lacasta, F. Lacava, J. Lacey, H. Lacker, D. Lacour, V. R. Lacuesta, E. Ladygin, R. Lafaye, B. Laforge, T. Lagouri, S. Lai, E. Laisne, L. Lambourne, C. L. Lampen, W. Lampl, E. Lancon, U. Landgraf, M. P. J. Landon, V. S. Lang, C. Lange, A. J. Lankford, F. Lanni, K. Lantzsch, A. Lanza, S. Laplace, C. Lapoire, J. F. Laporte, T. Lari, A. Larner, M. Lassnig, P. Laurelli, V. Lavorini, W. Lavrijsen, P. Laycock, O. Le Dortz, E. Le Guirriec, E. Le Menedeu, T. LeCompte, F. Ledroit-Guillon, H. Lee, J. S. H. Lee, S. C. Lee, L. Lee, M. Lefebvre, M. Legendre, F. Legger, C. Leggett, M. Lehmacher, G. Lehmann Miotto, A. G. Leister, M. A. L. Leite, R. Leitner, D. Lellouch, B. Lemmer, V. Lendermann, K. J. C. Leney, T. Lenz, G. Lenzen, B. Lenzi, K. Leonhardt, S. Leontsinis, F. Lepold, C. Leroy, J-R. Lessard, C. G. Lester, C. M. Lester, J. Levêque, D. Levin, L. J. Levinson, A. Lewis, G. H. Lewis, A. M. Leyko, M. Leyton, B. Li, B. Li, H. Li, H. L. Li, S. Li, X. Li, Z. Liang, H. Liao, B. Liberti, P. Lichard, K. Lie, W. Liebig, C. Limbach, A. Limosani, M. Limper, S. C. Lin, F. Linde, J. T. Linnemann, E. Lipeles, A. Lipniacka, T. M. Liss, D. Lissauer, A. Lister, A. M. Litke, D. Liu, J. B. Liu, L. Liu, M. Liu, Y. Liu, M. Livan, S. S. A. Livermore, A. Lleres, J. Llorente Merino, S. L. Lloyd, E. Lobodzinska, P. Loch, W. S. Lockman, T. Loddenkoetter, F. K. Loebinger, A. Loginov, C. W. Loh, T. Lohse, K. Lohwasser, M. Lokajicek, V. P. Lombardo, R. E. Long, L. Lopes, D. Lopez Mateos, J. Lorenz, N. Lorenzo Martinez, M. Losada, P. Loscutoff, F. Lo Sterzo, M. J. Losty, X. Lou, A. Lounis, K. F. Loureiro, J. Love, P. A. Love, A. J. Lowe, F. Lu, H. J. Lubatti, C. Luci, A. Lucotte, D. Ludwig, I. Ludwig, J. Ludwig, F. Luehring, G. Luijckx, W. Lukas, L. Luminari, E. Lund, B. Lund-Jensen, B. Lundberg, J. Lundberg, O. Lundberg, J. Lundquist, M. Lungwitz, D. Lynn, E. Lytken, H. Ma, L. L. Ma, G. Maccarrone, A. Macchiolo, B. Maček, J. Machado Miguens, D. Macina, R. Mackeprang, R. J. Madaras, H. J. Maddocks, W. F. Mader, M. Maeno, T. Maeno, P. Mättig, S. Mättig, L. Magnoni, E. Magradze, K. Mahboubi, J. Mahlstedt, S. Mahmoud, G. Mahout, C. Maiani, C. Maidantchik, A. Maio, S. Majewski, Y. Makida, N. Makovec, P. Mal, B. Malaescu, Pa. Malecki, P. Malecki, V. P. Maleev, F. Malek, U. Mallik, D. Malon, C. Malone, S. Maltezos, V. Malyshev, S. Malyukov, J. Mamuzic, A. Manabe, L. Mandelli, I. Mandić, R. Mandrysch, J. Maneira, A. Manfredini, L. Manhaes de Andrade Filho, J. A. Manjarres Ramos, A. Mann, P. M. Manning, A. Manousakis-Katsikakis, B. Mansoulie, R. Mantifel, A. Mapelli, L. Mapelli, L. March, J. F. Marchand, F. Marchese, G. Marchiori, M. Marcisovsky, C. P. Marino, F. Marroquim, Z. Marshall, L. F. Marti, S. Marti-Garcia, B. Martin, B. Martin, J. P. Martin, T. A. Martin, V. J. Martin, B. Martin dit Latour, S. Martin-Haugh, H. Martinez, M. Martinez, V. Martinez Outschoorn, A. C. Martyniuk, M. Marx, F. Marzano, A. Marzin, L. Masetti, T. Mashimo, R. Mashinistov, J. Masik, A. L. Maslennikov, I. Massa, G. Massaro, N. Massol, P. Mastrandrea, A. Mastroberardino, T. Masubuchi, H. Matsunaga, T. Matsushita, C. Mattravers, J. Maurer, S. J. Maxfield, D. A. Maximov, R. Mazini, M. Mazur, L. Mazzaferro, M. Mazzanti, J. Mc Donald, S. P. Mc Kee, A. McCarn, R. L. McCarthy, T. G. McCarthy, N. A. McCubbin, K. W. McFarlane, J. A. Mcfayden, G. Mchedlidze, T. Mclaughlan, S. J. McMahon, R. A. McPherson, A. Meade, J. Mechnich, M. Mechtel, M. Medinnis, S. Meehan, R. Meera-Lebbai, T. Meguro, S. Mehlhase, A. Mehta, K. Meier, B. Meirose, C. Melachrinos, B. R. Mellado Garcia, F. Meloni, L. Mendoza Navas, Z. Meng, A. Mengarelli, S. Menke, E. Meoni, K. M. Mercurio, P. Mermod, L. Merola, C. Meroni, F. S. Merritt, H. Merritt, A. Messina, J. Metcalfe, A. S. Mete, C. Meyer, C. Meyer, J-P. Meyer, J. Meyer, J. Meyer, S. Michal, L. Micu, R. P. Middleton, S. Migas, L. Mijović, G. Mikenberg, M. Mikestikova, M. Mikuž, D. W. Miller, R. J. Miller, W. J. Mills, C. Mills, A. Milov, D. A. Milstead, D. Milstein, A. A. Minaenko, M. Miñano Moya, I. A. Minashvili, A. I. Mincer, B. Mindur, M. Mineev, Y. Ming, L. M. Mir, G. Mirabelli, J. Mitrevski, V. A. Mitsou, S. Mitsui, P. S. Miyagawa, J. U. Mjörnmark, T. Moa, V. Moeller, K. Mönig, N. Möser, S. Mohapatra, W. Mohr, R. Moles-Valls, A. Molfetas, J. Monk, E. Monnier, J. Montejo Berlingen, F. Monticelli, S. Monzani, R. W. Moore, G. F. Moorhead, C. Mora Herrera, A. Moraes, N. Morange, J. Morel, G. Morello, D. Moreno, M. Moreno Llácer, P. Morettini, M. Morgenstern, M. Morii, A. K. Morley, G. Mornacchi, J. D. Morris, L. Morvaj, H. G. Moser, M. Mosidze, J. Moss, R. Mount, E. Mountricha, S. V. Mouraviev, E. J. W. Moyse, F. Mueller, J. Mueller, K. Mueller, T. A. Müller, T. Mueller, D. Muenstermann, Y. Munwes, W. J. Murray, I. Mussche, E. Musto, A. G. Myagkov, M. Myska, O. Nackenhorst, J. Nadal, K. Nagai, R. Nagai, Y. Nagai, K. Nagano, A. Nagarkar, Y. Nagasaka, M. Nagel, A. M. Nairz, Y. Nakahama, K. Nakamura, T. Nakamura, I. Nakano, G. Nanava, A. Napier, R. Narayan, M. Nash, T. Nattermann, T. Naumann, G. Navarro, H. A. Neal, P. Yu. Nechaeva, T. J. Neep, A. Negri, G. Negri, M. Negrini, S. Nektarijevic, A. Nelson, T. K. Nelson, S. Nemecek, P. Nemethy, A. A. Nepomuceno, M. Nessi, M. S. Neubauer, M. Neumann, A. Neusiedl, R. M. Neves, P. Nevski, F. M. Newcomer, P. R. Newman, V. Nguyen Thi Hong, R. B. Nickerson, R. Nicolaidou, B. Nicquevert, F. Niedercorn, J. Nielsen, N. Nikiforou, A. Nikiforov, V. Nikolaenko, I. Nikolic-Audit, K. Nikolics, K. Nikolopoulos, H. Nilsen, P. Nilsson, Y. Ninomiya, A. Nisati, R. Nisius, T. Nobe, L. Nodulman, M. Nomachi, I. Nomidis, S. Norberg, M. Nordberg, J. Novakova, M. Nozaki, L. Nozka, A.-E. Nuncio-Quiroz, G. Nunes Hanninger, T. Nunnemann, E. Nurse, B. J. O’Brien, D. C. O’Neil, V. O’Shea, L. B. Oakes, F. G. Oakham, H. Oberlack, J. Ocariz, A. Ochi, S. Oda, S. Odaka, J. Odier, H. Ogren, A. Oh, S. H. Oh, C. C. Ohm, T. Ohshima, W. Okamura, H. Okawa, Y. Okumura, T. Okuyama, A. Olariu, A. G. Olchevski, S. A. Olivares Pino, M. Oliveira, D. Oliveira Damazio, E. Oliver Garcia, D. Olivito, A. Olszewski, J. Olszowska, A. Onofre, P. U. E. Onyisi, C. J. Oram, M. J. Oreglia, Y. Oren, D. Orestano, N. Orlando, C. Oropeza Barrera, R. S. Orr, B. Osculati, R. Ospanov, C. Osuna, G. Otero y Garzon, J. P. Ottersbach, M. Ouchrif, E. A. Ouellette, F. Ould-Saada, A. Ouraou, Q. Ouyang, A. Ovcharova, M. Owen, S. Owen, V. E. Ozcan, N. Ozturk, A. Pacheco Pages, C. Padilla Aranda, S. Pagan Griso, E. Paganis, C. Pahl, F. Paige, P. Pais, K. Pajchel, G. Palacino, C. P. Paleari, S. Palestini, D. Pallin, A. Palma, J. D. Palmer, Y. B. Pan, E. Panagiotopoulou, J. G. Panduro Vazquez, P. Pani, N. Panikashvili, S. Panitkin, D. Pantea, A. Papadelis, Th. D. Papadopoulou, A. Paramonov, D. Paredes Hernandez, W. Park, M. A. Parker, F. Parodi, J. A. Parsons, U. Parzefall, S. Pashapour, E. Pasqualucci, S. Passaggio, A. Passeri, F. Pastore, Fr. Pastore, G. Pásztor, S. Pataraia, N. Patel, J. R. Pater, S. Patricelli, T. Pauly, S. Pedraza Lopez, M. I. Pedraza Morales, S. V. Peleganchuk, D. Pelikan, H. Peng, B. Penning, A. Penson, J. Penwell, M. Perantoni, K. Perez, T. Perez Cavalcanti, E. Perez Codina, M. T. Pérez García-Estañ, V. Perez Reale, L. Perini, H. Pernegger, R. Perrino, P. Perrodo, V. D. Peshekhonov, K. Peters, B. A. Petersen, J. Petersen, T. C. Petersen, E. Petit, A. Petridis, C. Petridou, E. Petrolo, F. Petrucci, D. Petschull, M. Petteni, R. Pezoa, A. Phan, P. W. Phillips, G. Piacquadio, A. Picazio, E. Piccaro, M. Piccinini, S. M. Piec, R. Piegaia, D. T. Pignotti, J. E. Pilcher, A. D. Pilkington, J. Pina, M. Pinamonti, A. Pinder, J. L. Pinfold, A. Pingel, B. Pinto, C. Pizio, M.-A. Pleier, E. Plotnikova, A. Poblaguev, S. Poddar, F. Podlyski, L. Poggioli, D. Pohl, M. Pohl, G. Polesello, A. Policicchio, R. Polifka, A. Polini, J. Poll, V. Polychronakos, D. Pomeroy, K. Pommès, L. Pontecorvo, B. G. Pope, G. A. Popeneciu, D. S. Popovic, A. Poppleton, X. Portell Bueso, G. E. Pospelov, S. Pospisil, I. N. Potrap, C. J. Potter, C. T. Potter, G. Poulard, J. Poveda, V. Pozdnyakov, R. Prabhu, P. Pralavorio, A. Pranko, S. Prasad, R. Pravahan, S. Prell, K. Pretzl, D. Price, J. Price, L. E. Price, D. Prieur, M. Primavera, K. Prokofiev, F. Prokoshin, S. Protopopescu, J. Proudfoot, X. Prudent, M. Przybycien, H. Przysiezniak, S. Psoroulas, E. Ptacek, E. Pueschel, D. Puldon, J. Purdham, M. Purohit, P. Puzo, Y. Pylypchenko, J. Qian, A. Quadt, D. R. Quarrie, W. B. Quayle, M. Raas, V. Radeka, V. Radescu, P. Radloff, F. Ragusa, G. Rahal, A. M. Rahimi, D. Rahm, S. Rajagopalan, M. Rammensee, M. Rammes, A. S. Randle-Conde, K. Randrianarivony, K. Rao, F. Rauscher, T. C. Rave, M. Raymond, A. L. Read, D. M. Rebuzzi, A. Redelbach, G. Redlinger, R. Reece, K. Reeves, A. Reinsch, I. Reisinger, C. Rembser, Z. L. Ren, A. Renaud, M. Rescigno, S. Resconi, B. Resende, P. Reznicek, R. Rezvani, R. Richter, E. Richter-Was, M. Ridel, M. Rijssenbeek, A. Rimoldi, L. Rinaldi, R. R. Rios, E. Ritsch, I. Riu, G. Rivoltella, F. Rizatdinova, E. Rizvi, S. H. Robertson, A. Robichaud-Veronneau, D. Robinson, J. E. M. Robinson, A. Robson, J. G. Rocha de Lima, C. Roda, D. Roda Dos Santos, A. Roe, S. Roe, O. Røhne, S. Rolli, A. Romaniouk, M. Romano, G. Romeo, E. Romero Adam, N. Rompotis, L. Roos, E. Ros, S. Rosati, K. Rosbach, A. Rose, M. Rose, G. A. Rosenbaum, P. L. Rosendahl, O. Rosenthal, L. Rosselet, V. Rossetti, E. Rossi, L. P. Rossi, M. Rotaru, I. Roth, J. Rothberg, D. Rousseau, C. R. Royon, A. Rozanov, Y. Rozen, X. Ruan, F. Rubbo, I. Rubinskiy, N. Ruckstuhl, V. I. Rud, C. Rudolph, F. Rühr, A. Ruiz-Martinez, L. Rumyantsev, Z. Rurikova, N. A. Rusakovich, A. Ruschke, J. P. Rutherfoord, N. Ruthmann, P. Ruzicka, Y. F. Ryabov, M. Rybar, G. Rybkin, N. C. Ryder, A. F. Saavedra, I. Sadeh, H. F-W. Sadrozinski, R. Sadykov, F. Safai Tehrani, H. Sakamoto, G. Salamanna, A. Salamon, M. Saleem, D. Salek, D. Salihagic, A. Salnikov, J. Salt, B. M. Salvachua Ferrando, D. Salvatore, F. Salvatore, A. Salvucci, A. Salzburger, D. Sampsonidis, B. H. Samset, A. Sanchez, V. Sanchez Martinez, H. Sandaker, H. G. Sander, M. P. Sanders, M. Sandhoff, T. Sandoval, C. Sandoval, R. Sandstroem, D. P. C. Sankey, A. Sansoni, C. Santamarina Rios, C. Santoni, R. Santonico, H. Santos, I. Santoyo Castillo, J. G. Saraiva, T. Sarangi, E. Sarkisyan-Grinbaum, B. Sarrazin, F. Sarri, G. Sartisohn, O. Sasaki, Y. Sasaki, N. Sasao, I. Satsounkevitch, G. Sauvage, E. Sauvan, J. B. Sauvan, P. Savard, V. Savinov, D. O. Savu, L. Sawyer, D. H. Saxon, J. Saxon, C. Sbarra, A. Sbrizzi, D. A. Scannicchio, M. Scarcella, J. Schaarschmidt, P. Schacht, D. Schaefer, U. Schäfer, A. Schaelicke, S. Schaepe, S. Schaetzel, A. C. Schaffer, D. Schaile, R. D. Schamberger, V. Scharf, V. A. Schegelsky, D. Scheirich, M. Schernau, M. I. Scherzer, C. Schiavi, J. Schieck, M. Schioppa, S. Schlenker, E. Schmidt, K. Schmieden, C. Schmitt, S. Schmitt, B. Schneider, Y. J. Schnellbach, U. Schnoor, L. Schoeffel, A. Schoening, A. L. S. Schorlemmer, M. Schott, D. Schouten, J. Schovancova, M. Schram, C. Schroeder, N. Schroer, M. J. Schultens, J. Schultes, H.-C. Schultz-Coulon, H. Schulz, M. Schumacher, B. A. Schumm, Ph. Schune, A. Schwartzman, Ph. Schwegler, Ph. Schwemling, R. Schwienhorst, J. Schwindling, T. Schwindt, M. Schwoerer, F. G. Sciacca, E. Scifo, G. Sciolla, W. G. Scott, J. Searcy, G. Sedov, E. Sedykh, S. C. Seidel, A. Seiden, F. Seifert, J. M. Seixas, G. Sekhniaidze, S. J. Sekula, K. E. Selbach, D. M. Seliverstov, B. Sellden, G. Sellers, M. Seman, N. Semprini-Cesari, C. Serfon, L. Serin, L. Serkin, T. Serre, R. Seuster, H. Severini, A. Sfyrla, E. Shabalina, M. Shamim, L. Y. Shan, J. T. Shank, Q. T. Shao, M. Shapiro, P. B. Shatalov, K. Shaw, D. Sherman, P. Sherwood, S. Shimizu, M. Shimojima, T. Shin, M. Shiyakova, A. Shmeleva, M. J. Shochet, D. Short, S. Shrestha, E. Shulga, M. A. Shupe, P. Sicho, A. Sidoti, F. Siegert, Dj. Sijacki, O. Silbert, J. Silva, Y. Silver, D. Silverstein, S. B. Silverstein, V. Simak, O. Simard, Lj. Simic, S. Simion, E. Simioni, B. Simmons, R. Simoniello, M. Simonyan, P. Sinervo, N. B. Sinev, V. Sipica, G. Siragusa, A. Sircar, A. N. Sisakyan, S. Yu. Sivoklokov, J. Sjölin, T. B. Sjursen, L. A. Skinnari, H. P. Skottowe, K. Skovpen, P. Skubic, M. Slater, T. Slavicek, K. Sliwa, V. Smakhtin, B. H. Smart, L. Smestad, S. Yu. Smirnov, Y. Smirnov, L. N. Smirnova, O. Smirnova, B. C. Smith, K. M. Smith, M. Smizanska, K. Smolek, A. A. Snesarev, G. Snidero, S. W. Snow, J. Snow, S. Snyder, R. Sobie, J. Sodomka, A. Soffer, C. A. Solans, M. Solar, J. Solc, E. Yu. Soldatov, U. Soldevila, E. Solfaroli Camillocci, A. A. Solodkov, O. V. Solovyanov, V. Solovyev, N. Soni, A. Sood, V. Sopko, B. Sopko, M. Sosebee, R. Soualah, P. Soueid, A. Soukharev, D. South, S. Spagnolo, F. Spanò, R. Spighi, G. Spigo, R. Spiwoks, M. Spousta, T. Spreitzer, B. Spurlock, R. D. St. Denis, J. Stahlman, R. Stamen, E. Stanecka, R. W. Stanek, C. Stanescu, M. Stanescu-Bellu, M. M. Stanitzki, S. Stapnes, E. A. Starchenko, J. Stark, P. Staroba, P. Starovoitov, R. Staszewski, A. Staude, P. Stavina, G. Steele, P. Steinbach, P. Steinberg, I. Stekl, B. Stelzer, H. J. Stelzer, O. Stelzer-Chilton, H. Stenzel, S. Stern, G. A. Stewart, J. A. Stillings, M. C. Stockton, M. Stoebe, K. Stoerig, G. Stoicea, S. Stonjek, P. Strachota, A. R. Stradling, A. Straessner, J. Strandberg, S. Strandberg, A. Strandlie, M. Strang, E. Strauss, M. Strauss, P. Strizenec, R. Ströhmer, D. M. Strom, J. A. Strong, R. Stroynowski, B. Stugu, I. Stumer, J. Stupak, P. Sturm, N. A. Styles, D. A. Soh, D. Su, HS. Subramania, R. Subramaniam, A. Succurro, Y. Sugaya, C. Suhr, M. Suk, V. V. Sulin, S. Sultansoy, T. Sumida, X. Sun, J. E. Sundermann, K. Suruliz, G. Susinno, M. R. Sutton, Y. Suzuki, Y. Suzuki, M. Svatos, S. Swedish, I. Sykora, T. Sykora, J. Sánchez, D. Ta, K. Tackmann, A. Taffard, R. Tafirout, N. Taiblum, Y. Takahashi, H. Takai, R. Takashima, H. Takeda, T. Takeshita, Y. Takubo, M. Talby, A. Talyshev, M. C. Tamsett, K. G. Tan, J. Tanaka, R. Tanaka, S. Tanaka, S. Tanaka, A. J. Tanasijczuk, K. Tani, N. Tannoury, S. Tapprogge, D. Tardif, S. Tarem, F. Tarrade, G. F. Tartarelli, P. Tas, M. Tasevsky, E. Tassi, Y. Tayalati, C. Taylor, F. E. Taylor, G. N. Taylor, W. Taylor, M. Teinturier, F. A. Teischinger, M. Teixeira Dias Castanheira, P. Teixeira-Dias, K. K. Temming, H. Ten Kate, P. K. Teng, S. Terada, K. Terashi, J. Terron, M. Testa, R. J. Teuscher, J. Therhaag, T. Theveneaux-Pelzer, S. Thoma, J. P. Thomas, E. N. Thompson, P. D. Thompson, P. D. Thompson, A. S. Thompson, L. A. Thomsen, E. Thomson, M. Thomson, W. M. Thong, R. P. Thun, F. Tian, M. J. Tibbetts, T. Tic, V. O. Tikhomirov, Y. A. Tikhonov, S. Timoshenko, E. Tiouchichine, P. Tipton, S. Tisserant, T. Todorov, S. Todorova-Nova, B. Toggerson, J. Tojo, S. Tokár, K. Tokushuku, K. Tollefson, M. Tomoto, L. Tompkins, K. Toms, A. Tonoyan, C. Topfel, N. D. Topilin, E. Torrence, H. Torres, E. Torró Pastor, J. Toth, F. Touchard, D. R. Tovey, T. Trefzger, L. Tremblet, A. Tricoli, I. M. Trigger, S. Trincaz-Duvoid, M. F. Tripiana, N. Triplett, W. Trischuk, B. Trocmé, C. Troncon, M. Trottier-McDonald, P. True, M. Trzebinski, A. Trzupek, C. Tsarouchas, J. C-L. Tseng, M. Tsiakiris, P. V. Tsiareshka, D. Tsionou, G. Tsipolitis, S. Tsiskaridze, V. Tsiskaridze, E. G. Tskhadadze, I. I. Tsukerman, V. Tsulaia, J.-W. Tsung, S. Tsuno, D. Tsybychev, A. Tua, A. Tudorache, V. Tudorache, J. M. Tuggle, M. Turala, D. Turecek, I. Turk Cakir, R. Turra, P. M. Tuts, A. Tykhonov, M. Tylmad, M. Tyndel, G. Tzanakos, K. Uchida, I. Ueda, R. Ueno, M. Ughetto, M. Ugland, M. Uhlenbrock, F. Ukegawa, G. Unal, A. Undrus, G. Unel, Y. Unno, D. Urbaniec, P. Urquijo, G. Usai, L. Vacavant, V. Vacek, B. Vachon, S. Vahsen, S. Valentinetti, A. Valero, L. Valery, S. Valkar, E. Valladolid Gallego, S. Vallecorsa, J. A. Valls Ferrer, R. Van Berg, P. C. Van Der Deijl, R. van der Geer, H. van der Graaf, R. Van Der Leeuw, E. van der Poel, D. van der Ster, N. van Eldik, P. van Gemmeren, J. Van Nieuwkoop, I. van Vulpen, M. Vanadia, W. Vandelli, A. Vaniachine, P. Vankov, F. Vannucci, R. Vari, E. W. Varnes, T. Varol, D. Varouchas, A. Vartapetian, K. E. Varvell, V. I. Vassilakopoulos, F. Vazeille, T. Vazquez Schroeder, G. Vegni, J. J. Veillet, F. Veloso, R. Veness, S. Veneziano, A. Ventura, D. Ventura, M. Venturi, N. Venturi, V. Vercesi, M. Verducci, W. Verkerke, J. C. Vermeulen, A. Vest, M. C. Vetterli, I. Vichou, T. Vickey, O. E. Vickey Boeriu, G. H. A. Viehhauser, S. Viel, M. Villa, M. Villaplana Perez, E. Vilucchi, M. G. Vincter, E. Vinek, V. B. Vinogradov, M. Virchaux, J. Virzi, O. Vitells, M. Viti, I. Vivarelli, F. Vives Vaque, S. Vlachos, D. Vladoiu, M. Vlasak, A. Vogel, P. Vokac, G. Volpi, M. Volpi, G. Volpini, H. von der Schmitt, H. von Radziewski, E. von Toerne, V. Vorobel, V. Vorwerk, M. Vos, R. Voss, J. H. Vossebeld, N. Vranjes, M. Vranjes Milosavljevic, V. Vrba, M. Vreeswijk, T. Vu Anh, R. Vuillermet, I. Vukotic, W. Wagner, P. Wagner, H. Wahlen, S. Wahrmund, J. Wakabayashi, S. Walch, J. Walder, R. Walker, W. Walkowiak, R. Wall, P. Waller, B. Walsh, C. Wang, H. Wang, H. Wang, J. Wang, J. Wang, R. Wang, S. M. Wang, T. Wang, A. Warburton, C. P. Ward, D. R. Wardrope, M. Warsinsky, A. Washbrook, C. Wasicki, I. Watanabe, P. M. Watkins, A. T. Watson, I. J. Watson, M. F. Watson, G. Watts, S. Watts, A. T. Waugh, B. M. Waugh, M. S. Weber, J. S. Webster, A. R. Weidberg, P. Weigell, J. Weingarten, C. Weiser, P. S. Wells, T. Wenaus, D. Wendland, Z. Weng, T. Wengler, S. Wenig, N. Wermes, M. Werner, P. Werner, M. Werth, M. Wessels, J. Wetter, C. Weydert, K. Whalen, A. White, M. J. White, S. White, S. R. Whitehead, D. Whiteson, D. Whittington, D. Wicke, F. J. Wickens, W. Wiedenmann, M. Wielers, P. Wienemann, C. Wiglesworth, L. A. M. Wiik-Fuchs, P. A. Wijeratne, A. Wildauer, M. A. Wildt, I. Wilhelm, H. G. Wilkens, J. Z. Will, E. Williams, H. H. Williams, S. Williams, W. Willis, S. Willocq, J. A. Wilson, M. G. Wilson, A. Wilson, I. Wingerter-Seez, S. Winkelmann, F. Winklmeier, M. Wittgen, S. J. Wollstadt, M. W. Wolter, H. Wolters, W. C. Wong, G. Wooden, B. K. Wosiek, J. Wotschack, M. J. Woudstra, K. W. Wozniak, K. Wraight, M. Wright, B. Wrona, S. L. Wu, X. Wu, Y. Wu, E. Wulf, B. M. Wynne, S. Xella, M. Xiao, S. Xie, C. Xu, D. Xu, L. Xu, B. Yabsley, S. Yacoob, M. Yamada, H. Yamaguchi, A. Yamamoto, K. Yamamoto, S. Yamamoto, T. Yamamura, T. Yamanaka, K. Yamauchi, T. Yamazaki, Y. Yamazaki, Z. Yan, H. Yang, H. Yang, U. K. Yang, Y. Yang, Z. Yang, S. Yanush, L. Yao, Y. Yasu, E. Yatsenko, J. Ye, S. Ye, A. L. Yen, M. Yilmaz, R. Yoosoofmiya, K. Yorita, R. Yoshida, K. Yoshihara, C. Young, C. J. Young, S. Youssef, D. Yu, D. R. Yu, J. Yu, J. Yu, L. Yuan, A. Yurkewicz, B. Zabinski, R. Zaidan, A. M. Zaitsev, L. Zanello, D. Zanzi, A. Zaytsev, C. Zeitnitz, M. Zeman, A. Zemla, O. Zenin, T. Ženiš, Z. Zinonos, D. Zerwas, G. Zevi della Porta, D. Zhang, H. Zhang, J. Zhang, X. Zhang, Z. Zhang, L. Zhao, Z. Zhao, A. Zhemchugov, J. Zhong, B. Zhou, N. Zhou, Y. Zhou, C. G. Zhu, H. Zhu, J. Zhu, Y. Zhu, X. Zhuang, V. Zhuravlov, A. Zibell, D. Zieminska, N. I. Zimin, R. Zimmermann, S. Zimmermann, S. Zimmermann, M. Ziolkowski, R. Zitoun, L. Živković, V. V. Zmouchko, G. Zobernig, A. Zoccoli, M. zur Nedden, V. Zutshi, L. Zwalinski

**Affiliations:** 1 CERN, 1211 Geneva 23, Switzerland; 2 School of Chemistry and Physics, University of Adelaide, Adelaide, Australia; 3 Physics Department, SUNY Albany, Albany, NY United States of America; 4 Department of Physics, University of Alberta, Edmonton, AB Canada; 5 Department of Physics, Ankara University, Ankara, Turkey; 6 Department of Physics, Gazi University, Ankara, Turkey; 7 Division of Physics, TOBB University of Economics and Technology, Ankara, Turkey; 8 Turkish Atomic Energy Authority, Ankara, Turkey; 9 LAPP, CNRS/IN2P3 and Université de Savoie, Annecy-le-Vieux, France; 10 High Energy Physics Division, Argonne National Laboratory, Argonne, IL United States of America; 11 Department of Physics, University of Arizona, Tucson, AZ United States of America; 12 Department of Physics, The University of Texas at Arlington, Arlington, TX United States of America; 13 Physics Department, University of Athens, Athens, Greece; 14 Physics Department, National Technical University of Athens, Zografou, Greece; 15 Institute of Physics, Azerbaijan Academy of Sciences, Baku, Azerbaijan; 16 Institut de Física d’Altes Energies and Departament de Física de la Universitat Autònoma de Barcelona and ICREA, Barcelona, Spain; 17 Institute of Physics, University of Belgrade, Belgrade, Serbia; 18 Vinca Institute of Nuclear Sciences, University of Belgrade, Belgrade, Serbia; 19 Department for Physics and Technology, University of Bergen, Bergen, Norway; 20 Physics Division, Lawrence Berkeley National Laboratory and University of California, Berkeley, CA United States of America; 21 Department of Physics, Humboldt University, Berlin, Germany; 22 Albert Einstein Center for Fundamental Physics and Laboratory for High Energy Physics, University of Bern, Bern, Switzerland; 23 School of Physics and Astronomy, University of Birmingham, Birmingham, United Kingdom; 24 Department of Physics, Bogazici University, Istanbul, Turkey; 25 Division of Physics, Dogus University, Istanbul, Turkey; 26 Department of Physics Engineering, Gaziantep University, Gaziantep, Turkey; 27 INFN Sezione di Bologna, Bologna, Italy; 28 Dipartimento di Fisica, Università di Bologna, Bologna, Italy; 29 Physikalisches Institut, University of Bonn, Bonn, Germany; 30 Department of Physics, Boston University, Boston, MA United States of America; 31 Department of Physics, Brandeis University, Waltham, MA United States of America; 32 Universidade Federal do Rio De Janeiro COPPE/EE/IF, Rio de Janeiro, Brazil; 33 Federal University of Juiz de Fora (UFJF), Juiz de Fora, Brazil; 34 Federal University of Sao Joao del Rei (UFSJ), Sao Joao del Rei, Brazil; 35 Instituto de Fisica, Universidade de Sao Paulo, Sao Paulo, Brazil; 36 Physics Department, Brookhaven National Laboratory, Upton, NY United States of America; 37 National Institute of Physics and Nuclear Engineering, Bucharest, Romania; 38 University Politehnica Bucharest, Bucharest, Romania; 39 West University in Timisoara, Timisoara, Romania; 40 Departamento de Física, Universidad de Buenos Aires, Buenos Aires, Argentina; 41 Cavendish Laboratory, University of Cambridge, Cambridge, United Kingdom; 42 Department of Physics, Carleton University, Ottawa, ON Canada; 43 CERN, Geneva, Switzerland; 44 Enrico Fermi Institute, University of Chicago, Chicago, IL United States of America; 45 Departamento de Física, Pontificia Universidad Católica de Chile, Santiago, Chile; 46 Departamento de Física, Universidad Técnica Federico Santa María, Valparaíso, Chile; 47 Institute of High Energy Physics, Chinese Academy of Sciences, Beijing, China; 48 Department of Modern Physics, University of Science and Technology of China, Anhui, China; 49 Department of Physics, Nanjing University, Jiangsu, China; 50 School of Physics, Shandong University, Shandong, China; 51 Physics Department, Shanghai Jiao Tong University, Shanghai, China; 52 Laboratoire de Physique Corpusculaire, Clermont Université and Université Blaise Pascal and CNRS/IN2P3, Clermont-Ferrand, France; 53 Nevis Laboratory, Columbia University, Irvington, NY United States of America; 54 Niels Bohr Institute, University of Copenhagen, Kobenhavn, Denmark; 55 INFN Gruppo Collegato di Cosenza, Arcavata di Rende, Italy; 56 Dipartimento di Fisica, Università della Calabria, Arcavata di Rende, Italy; 57 Faculty of Physics and Applied Computer Science, AGH University of Science and Technology, Krakow, Poland; 58 The Henryk Niewodniczanski Institute of Nuclear Physics, Polish Academy of Sciences, Krakow, Poland; 59 Physics Department, Southern Methodist University, Dallas, TX United States of America; 60 Physics Department, University of Texas at Dallas, Richardson, TX United States of America; 61 DESY, Hamburg and Zeuthen, Germany; 62 Institut für Experimentelle Physik IV, Technische Universität Dortmund, Dortmund, Germany; 63 Institut für Kern- und Teilchenphysik, Technical University Dresden, Dresden, Germany; 64 Department of Physics, Duke University, Durham, NC United States of America; 65 SUPA - School of Physics and Astronomy, University of Edinburgh, Edinburgh, United Kingdom; 66 INFN Laboratori Nazionali di Frascati, Frascati, Italy; 67 Fakultät für Mathematik und Physik, Albert-Ludwigs-Universität, Freiburg, Germany; 68 Section de Physique, Université de Genève, Geneva, Switzerland; 69 INFN Sezione di Genova, Genova, Italy; 70 Dipartimento di Fisica, Università di Genova, Genova, Italy; 71 E. Andronikashvili Institute of Physics, Iv. Javakhishvili Tbilisi State University, Tbilisi, Georgia; 72 High Energy Physics Institute, Tbilisi State University, Tbilisi, Georgia; 73 II Physikalisches Institut, Justus-Liebig-Universität Giessen, Giessen, Germany; 74 SUPA - School of Physics and Astronomy, University of Glasgow, Glasgow, United Kingdom; 75 II Physikalisches Institut, Georg-August-Universität, Göttingen, Germany; 76 Laboratoire de Physique Subatomique et de Cosmologie, Université Joseph Fourier and CNRS/IN2P3 and Institut National Polytechnique de Grenoble, Grenoble, France; 77 Department of Physics, Hampton University, Hampton, VA United States of America; 78 Laboratory for Particle Physics and Cosmology, Harvard University, Cambridge, MA United States of America; 79 Kirchhoff-Institut für Physik, Ruprecht-Karls-Universität Heidelberg, Heidelberg, Germany; 80 Physikalisches Institut, Ruprecht-Karls-Universität Heidelberg, Heidelberg, Germany; 81 ZITI Institut für technische Informatik, Ruprecht-Karls-Universität Heidelberg, Mannheim, Germany; 82 Faculty of Applied Information Science, Hiroshima Institute of Technology, Hiroshima, Japan; 83 Department of Physics, Indiana University, Bloomington, IN United States of America; 84 Institut für Astro- und Teilchenphysik, Leopold-Franzens-Universität, Innsbruck, Austria; 85 University of Iowa, Iowa City, IA United States of America; 86 Department of Physics and Astronomy, Iowa State University, Ames, IA United States of America; 87 Joint Institute for Nuclear Research, JINR Dubna, Dubna, Russia; 88 KEK, High Energy Accelerator Research Organization, Tsukuba, Japan; 89 Graduate School of Science, Kobe University, Kobe, Japan; 90 Faculty of Science, Kyoto University, Kyoto, Japan; 91 Kyoto University of Education, Kyoto, Japan; 92 Department of Physics, Kyushu University, Fukuoka, Japan; 93 Instituto de Física La Plata, Universidad Nacional de La Plata and CONICET, La Plata, Argentina; 94 Physics Department, Lancaster University, Lancaster, United Kingdom; 95 INFN Sezione di Lecce, Lecce, Italy; 96 Dipartimento di Matematica e Fisica, Università del Salento, Lecce, Italy; 97 Oliver Lodge Laboratory, University of Liverpool, Liverpool, United Kingdom; 98 Department of Physics, Jožef Stefan Institute and University of Ljubljana, Ljubljana, Slovenia; 99 School of Physics and Astronomy, Queen Mary University of London, London, United Kingdom; 100 Department of Physics, Royal Holloway University of London, Surrey, United Kingdom; 101 Department of Physics and Astronomy, University College London, London, United Kingdom; 102 Laboratoire de Physique Nucléaire et de Hautes Energies, UPMC and Université Paris-Diderot and CNRS/IN2P3, Paris, France; 103 Fysiska institutionen, Lunds universitet, Lund, Sweden; 104 Departamento de Fisica Teorica C-15, Universidad Autonoma de Madrid, Madrid, Spain; 105 Institut für Physik, Universität Mainz, Mainz, Germany; 106 School of Physics and Astronomy, University of Manchester, Manchester, United Kingdom; 107 CPPM, Aix-Marseille Université and CNRS/IN2P3, Marseille, France; 108 Department of Physics, University of Massachusetts, Amherst, MA United States of America; 109 Department of Physics, McGill University, Montreal, QC Canada; 110 School of Physics, University of Melbourne, Victoria, Australia; 111 Department of Physics, The University of Michigan, Ann Arbor, MI United States of America; 112 Department of Physics and Astronomy, Michigan State University, East Lansing, MI United States of America; 113 INFN Sezione di Milano, Milano, Italy; 114 Dipartimento di Fisica, Università di Milano, Milano, Italy; 115 B.I. Stepanov Institute of Physics, National Academy of Sciences of Belarus, Minsk, Republic of Belarus; 116 National Scientific and Educational Centre for Particle and High Energy Physics, Minsk, Republic of Belarus; 117 Department of Physics, Massachusetts Institute of Technology, Cambridge, MA United States of America; 118 Group of Particle Physics, University of Montreal, Montreal, QC Canada; 119 P.N. Lebedev Institute of Physics, Academy of Sciences, Moscow, Russia; 120 Institute for Theoretical and Experimental Physics (ITEP), Moscow, Russia; 121 Moscow Engineering and Physics Institute (MEPhI), Moscow, Russia; 122 D.V.Skobeltsyn Institute of Nuclear Physics, M.V.Lomonosov Moscow State University, Moscow, Russia; 123 Fakultät für Physik, Ludwig-Maximilians-Universität München, München, Germany; 124 Max-Planck-Institut für Physik (Werner-Heisenberg-Institut), München, Germany; 125 Nagasaki Institute of Applied Science, Nagasaki, Japan; 126 Graduate School of Science and Kobayashi-Maskawa Institute, Nagoya University, Nagoya, Japan; 127 INFN Sezione di Napoli, Napoli, Italy; 128 Dipartimento di Scienze Fisiche, Università di Napoli, Napoli, Italy; 129 Department of Physics and Astronomy, University of New Mexico, Albuquerque, NM United States of America; 130 Institute for Mathematics, Astrophysics and Particle Physics, Radboud University Nijmegen/Nikhef, Nijmegen, Netherlands; 131 Nikhef National Institute for Subatomic Physics and University of Amsterdam, Amsterdam, Netherlands; 132 Department of Physics, Northern Illinois University, DeKalb, IL United States of America; 133 Budker Institute of Nuclear Physics, SB RAS, Novosibirsk, Russia; 134 Department of Physics, New York University, New York, NY United States of America; 135 Ohio State University, Columbus, OH United States of America; 136 Faculty of Science, Okayama University, Okayama, Japan; 137 Homer L. Dodge Department of Physics and Astronomy, University of Oklahoma, Norman, OK United States of America; 138 Department of Physics, Oklahoma State University, Stillwater, OK United States of America; 139 RCPTM, Palacký University, Olomouc, Czech Republic; 140 Center for High Energy Physics, University of Oregon, Eugene, OR United States of America; 141 LAL, Université Paris-Sud and CNRS/IN2P3, Orsay, France; 142 Graduate School of Science, Osaka University, Osaka, Japan; 143 Department of Physics, University of Oslo, Oslo, Norway; 144 Department of Physics, Oxford University, Oxford, United Kingdom; 145 INFN Sezione di Pavia, Pavia, Italy; 146 Dipartimento di Fisica, Università di Pavia, Pavia, Italy; 147 Department of Physics, University of Pennsylvania, Philadelphia, PA United States of America; 148 Petersburg Nuclear Physics Institute, Gatchina, Russia; 149 INFN Sezione di Pisa, Pisa, Italy; 150 Dipartimento di Fisica E. Fermi, Università di Pisa, Pisa, Italy; 151 Department of Physics and Astronomy, University of Pittsburgh, Pittsburgh, PA United States of America; 152 Laboratorio de Instrumentacao e Fisica Experimental de Particulas - LIP, Lisboa, Portugal; 153 Departamento de Fisica Teorica y del Cosmos and CAFPE, Universidad de Granada, Granada, Spain; 154 Institute of Physics, Academy of Sciences of the Czech Republic, Praha, Czech Republic; 155 Czech Technical University in Prague, Praha, Czech Republic; 156 Faculty of Mathematics and Physics, Charles University in Prague, Praha, Czech Republic; 157 State Research Center Institute for High Energy Physics, Protvino, Russia; 158 Particle Physics Department, Rutherford Appleton Laboratory, Didcot, United Kingdom; 159 Physics Department, University of Regina, Regina, SK Canada; 160 Ritsumeikan University, Kusatsu, Shiga Japan; 161 INFN Sezione di Roma I, Roma, Italy; 162 Dipartimento di Fisica, Università La Sapienza, Roma, Italy; 163 INFN Sezione di Roma Tor Vergata, Roma, Italy; 164 Dipartimento di Fisica, Università di Roma Tor Vergata, Roma, Italy; 165 INFN Sezione di Roma Tre, Roma, Italy; 166 Dipartimento di Fisica, Università Roma Tre, Roma, Italy; 167 Faculté des Sciences Ain Chock, Réseau Universitaire de Physique des Hautes Energies - Université Hassan II, Casablanca, Morocco; 168 Centre National de l’Energie des Sciences Techniques Nucleaires, Rabat, Morocco; 169 Faculté des Sciences Semlalia, Université Cadi Ayyad, LPHEA, Marrakech, Morocco; 170 Faculté des Sciences, Université Mohamed Premier and LPTPM, Oujda, Morocco; 171 Faculté des sciences, Université Mohammed V-Agdal, Rabat, Morocco; 172 DSM/IRFU (Institut de Recherches sur les Lois Fondamentales de l’Univers), CEA Saclay (Commissariat à l’Energie Atomique et aux Energies Alternatives), Gif-sur-Yvette, France; 173 Santa Cruz Institute for Particle Physics, University of California Santa Cruz, Santa Cruz, CA United States of America; 174 Department of Physics, University of Washington, Seattle, WA United States of America; 175 Department of Physics and Astronomy, University of Sheffield, Sheffield, United Kingdom; 176 Department of Physics, Shinshu University, Nagano, Japan; 177 Fachbereich Physik, Universität Siegen, Siegen, Germany; 178 Department of Physics, Simon Fraser University, Burnaby, BC Canada; 179 SLAC National Accelerator Laboratory, Stanford, CA United States of America; 180 Faculty of Mathematics, Physics & Informatics, Comenius University, Bratislava, Slovak Republic; 181 Department of Subnuclear Physics, Institute of Experimental Physics of the Slovak Academy of Sciences, Kosice, Slovak Republic; 182 Department of Physics, University of Johannesburg, Johannesburg, South Africa; 183 School of Physics, University of the Witwatersrand, Johannesburg, South Africa; 184 Department of Physics, Stockholm University, Stockholm, Sweden; 185 The Oskar Klein Centre, Stockholm, Sweden; 186 Physics Department, Royal Institute of Technology, Stockholm, Sweden; 187 Departments of Physics & Astronomy and Chemistry, Stony Brook University, Stony Brook, NY United States of America; 188 Department of Physics and Astronomy, University of Sussex, Brighton, United Kingdom; 189 School of Physics, University of Sydney, Sydney, Australia; 190 Institute of Physics, Academia Sinica, Taipei, Taiwan; 191 Department of Physics, Technion: Israel Institute of Technology, Haifa, Israel; 192 Raymond and Beverly Sackler School of Physics and Astronomy, Tel Aviv University, Tel Aviv, Israel; 193 Department of Physics, Aristotle University of Thessaloniki, Thessaloniki, Greece; 194 International Center for Elementary Particle Physics and Department of Physics, The University of Tokyo, Tokyo, Japan; 195 Graduate School of Science and Technology, Tokyo Metropolitan University, Tokyo, Japan; 196 Department of Physics, Tokyo Institute of Technology, Tokyo, Japan; 197 Department of Physics, University of Toronto, Toronto, ON Canada; 198 TRIUMF, Vancouver, BC Canada; 199 Department of Physics and Astronomy, York University, Toronto, ON Canada; 200 Faculty of Pure and Applied Sciences, University of Tsukuba, Tsukuba, Japan; 201 Department of Physics and Astronomy, Tufts University, Medford, MA United States of America; 202 Centro de Investigaciones, Universidad Antonio Narino, Bogota, Colombia; 203 Department of Physics and Astronomy, University of California Irvine, Irvine, CA United States of America; 204 INFN Gruppo Collegato di Udine, Udine, Italy; 205 ICTP, Trieste, Italy; 206 Dipartimento di Chimica, Fisica e Ambiente, Università di Udine, Udine, Italy; 207 Department of Physics, University of Illinois, Urbana, IL United States of America; 208 Department of Physics and Astronomy, University of Uppsala, Uppsala, Sweden; 209 Instituto de Física Corpuscular (IFIC) and Departamento de Física Atómica, Molecular y Nuclear and Departamento de Ingeniería Electrónica and Instituto de Microelectrónica de Barcelona (IMB-CNM), University of Valencia and CSIC, Valencia, Spain; 210 Department of Physics, University of British Columbia, Vancouver, BC Canada; 211 Department of Physics and Astronomy, University of Victoria, Victoria, BC Canada; 212 Department of Physics, University of Warwick, Coventry, United Kingdom; 213 Waseda University, Tokyo, Japan; 214 Department of Particle Physics, The Weizmann Institute of Science, Rehovot, Israel; 215 Department of Physics, University of Wisconsin, Madison, WI United States of America; 216 Fakultät für Physik und Astronomie, Julius-Maximilians-Universität, Würzburg, Germany; 217 Fachbereich C Physik, Bergische Universität Wuppertal, Wuppertal, Germany; 218 Department of Physics, Yale University, New Haven, CT United States of America; 219 Yerevan Physics Institute, Yerevan, Armenia; 220 Centre de Calcul de l’Institut National de Physique Nucléaire et de Physique des Particules (IN2P3), Villeurbanne, France

## Abstract

A search for supersymmetric particles in final states with zero, one, and two leptons, with and without jets identified as originating from *b*-quarks, in 4.7 fb^−1^ of $\sqrt{s}=7\mbox{ TeV}$ *pp* collisions produced by the Large Hadron Collider and recorded by the ATLAS detector is presented. The search uses a set of variables carrying information on the event kinematics transverse and parallel to the beam line that are sensitive to several topologies expected in supersymmetry. Mutually exclusive final states are defined, allowing a combination of all channels to increase the search sensitivity. No deviation from the Standard Model expectation is observed. Upper limits at 95 % confidence level on visible cross-sections for the production of new particles are extracted. Results are interpreted in the context of the constrained minimal supersymmetric extension to the Standard Model and in supersymmetry-inspired models with diverse, high-multiplicity final states.

## Introduction

One of the most promising extensions of the Standard Model, supersymmetry (SUSY) [1–9], has been the target of a large number of searches at the LHC. Prompted by the large predicted production cross-section of coloured SUSY particles (sparticles), ATLAS and CMS have performed inclusive searches for strongly produced squarks and gluinos, the superpartners of quarks and gluons [10–17]. Assuming R-parity conservation [18–22], these sparticles are produced in pairs and decay into energetic jets, possibly leptons, and the lightest SUSY particle (LSP, typically the lightest neutralino $\tilde{\chi}_{1}^{0}$), which escapes detection and results in missing transverse momentum. For these searches, the selections adopted to discriminate the signal processes from the background typically include requirements on the missing transverse momentum ($E_{\mathrm{T}}^{\mathrm{miss}}$) and the scalar sum of transverse momenta of all selected physics objects (*H*_T_) plus the scalar $E_{\mathrm{T}}^{\mathrm{miss}}$ (effective mass, *M*_eff_).

This paper presents a search for strongly produced sparticles that makes use of a variety of final states including high transverse momentum jets and zero, one, or two leptons (electrons or muons). The events are also separated according to the presence of a jet identified as originating from a *b*-quark (*b*-tagged jet). Several mutually exclusive search channels are defined, facilitating a simultaneous search in all of the typical final states and increasing the search sensitivity. The search employs a set of observables, called the “razor variables” [23], which make use of both longitudinal and transverse event information. Because of the inclusion of longitudinal information, the requirements on the transverse information to reduce the background are effectively relaxed, making the search sensitive to different regions of kinematic phase space relative to other $E_{\mathrm{T}}^{\mathrm{miss}}$-based searches. Thus, these search results complement those already performed by ATLAS. These variables were first employed in SUSY searches by CMS [24, 25].

This paper is organised as follows. The main features of the ATLAS detector are presented in Sect. 2. Section 3 introduces the razor variables. Section 4 describes the data sample, basic event selection, and the Monte Carlo simulation used to model the data. Section 5 defines the basic physics objects and event-level variables that are used through the analysis. The search technique is described in Sect. 6, and the background estimation is presented in Sect. 7. The performance of the search and interpretation of the results are presented in Sect. 8. Finally, Sect. 9 includes a summary of the analysis and of its findings.

## ATLAS detector

The ATLAS detector comprises an inner tracking detector, a calorimeter, and a muon system [26]. The inner detector includes a silicon pixel detector, a silicon microstrip detector, and a transition radiation tracker. It is immersed in a 2 T axial field and precisely measures the tracks of charged particles in the pseudorapidity region^1^ |*η*|<2.5. The calorimeter covers the region |*η*|<4.9 and is divided into electromagnetic and hadronic compartments. The electromagnetic calorimetry in the central (|*η*|<3.2) region is provided by liquid argon sampling calorimeters with lead absorbers. In the barrel region (|*η*|<1.4), the hadronic calorimetry is provided by scintillator tiles with steel absorbers, and the more forward (1.4<|*η*|<3.2) region is covered by a liquid argon and copper sampling hadronic calorimeter. The forward calorimetry (|*η*|>3.2) uses liquid argon and copper or tungsten absorbers. The muon spectrometer covers |*η*|<2.7 and includes a system of air-core toroidal magnets. A variety of technologies are used to provide precision muon tracking and identification for |*η*|<2.7 and rapid response for triggering for |*η*|<2.4.

ATLAS uses a three-tier trigger system to select events. The first-level (L1) trigger is hardware-based and only uses coarse calorimeter information and muon system information. The calorimeter information available at the lowest level includes basic objects with rough calibration and simple identification of electromagnetic objects (electrons and photons) as distinct from hadronic objects (jets). The second-level (L2) trigger and event-filter (EF) compose the software-based high-level trigger (HLT), in which full event reconstruction is run, similar to that used offline, in order to accurately identify and measure objects. The L2 only examines *η*/*ϕ* regions that triggered the L1. The EF fully reconstructs events that pass L2.

## Razor variable definitions

Searches for sparticles in R-parity-conserving scenarios generally make the assumption that the sparticles are pair-produced and decay subsequently to an LSP that is invisible in the detector. The heavy sparticles produced are either the same type of particle (pair-production) or are at the same mass scale (i.e. scenarios with associated squark–gluino production are most relevant when *m*_squark_≈*m*_gluino_). Thus, the production mass and visible energy in the decays are fairly symmetric. Most analyses make use of the transverse balance of typical *pp* collision events, or exploit the event symmetry in the transverse plane. The razor variables attempt to also include longitudinal information about the event by making several assumptions motivated by the kinematics of the models of interest.

In the rest frame of each heavy sparticle, called the *R*-frame, the sparticle decays are symmetric. In an attempt to reconstruct the primary produced sparticle pair, the razor calculation clusters all final-state particles into a pair of objects with four-momenta called “mega-jets”. Each of these mega-jets is associated with one of the two SUSY decay chains and represents the visible energy-momentum of that produced sparticle. All possible combinations of the four-vectors of the visibly reconstructed/selected objects (signal jets and leptons) are considered when constructing the two mega-jets. The pair of mega-jets, *j*_1_ and *j*_2_, that minimises the sum of the squared masses of the four-vectors is selected. Following the prescription in Ref. [23] and for consistency with Ref. [24], all jets and the mega-jets are forced to be massless by setting their energy equal to the magnitude of their three momenta. Studies indicate that neither this choice nor the mega-jet selection, based on minimizing the mega-jet mass squared, have a significant impact on the reach of the razor-based search.

In the *R*-frames, each heavy sparticle should be nearly at rest with some mass *m*_Heavy_. The sparticle decay may then be approximated as a two-body decay to some visible object (a mega-jet) and the invisible, stable LSP. The final visible decay products (i.e. the final-state quarks and gluons, or the observable jets and leptons) have masses far below the SUSY mass scale and can therefore be approximated as being massless. Then the energy of each mega-jet in the *R*-frame, *E*_1_ and *E*_2_, becomes:

1 $$ E_1 = E_2 = \frac{ m_{\text{Heavy}}^2 - m_{\text{LSP}}^2 }{2 \times m_{\text{Heavy}}}, $$

where *m*_LSP_ is the mass of the LSP. This leads to a characteristic mass, *M*_*R*_, in the *R* frame of *M*_*R*_=2×*E*_1_=2×*E*_2_, which for *m*_Heavy_≫*m*_LSP_ is identical to *m*_Heavy_. Therefore, in events where heavy particles are pair-produced, *M*_*R*_, which is a measure of the scale of the heaviest particles produced, should form a bump [23, 24]. In $t\bar{t}$ or *WW* events, for example, the characteristic mass *M*_*R*_≈*m*_top_ or *m*_*W*_. Like the Jacobian peak of the transverse mass distribution in *W*→*ℓν* events, the width of the bump is dominated by the kinematics of the invisible particles in the event. The product of *M*_*R*_ and the Lorentz factor for the boost from the lab to *R*-frame, $M_{R}'=\gamma_{R} \times M_{R}$, is useful for characterisation of the sparticle mass scale, in part because of its close relation to *m*_Heavy_, and in part because Standard Model backgrounds tend to have small values of $M_{R}'$. When expressed in terms of the mega-jet quantities in the lab frame, the expression is given by:

2 $$ M_{R}' = \sqrt{ ( j_{1,E} + j_{2,E} )^2 - ( j_{1,z} + j_{2,z} )^2 } , $$

where *j*_*i*,*E*_ and *j*_*i*,*z*_ are the energy and longitudinal momentum, respectively, of mega-jet *i*. The transverse information of the system is taken into account by constructing a transverse mass for the mega-jets, assuming half of the $E_{\mathrm{T}}^{\mathrm{miss}}$ is associated with each jet: 3 where $\mathbf {E_{\mathrm{T}}^{\mathrm{miss}}}$ is the two-dimensional vector of the $E_{\mathrm{T}}^{\mathrm{miss}}$ in the transverse plane. When an event contains “fake” $E_{\mathrm{T}}^{\mathrm{miss}}$ from a detector defect or mismeasurement, the system will tend to have back-to-back mega-jets. In such cases, the vector sum of the two mega-jet momenta will be small. If, on the other hand, there is real $E_{\mathrm{T}}^{\mathrm{miss}}$, the mega-jets may not be back-to-back and may even point in the same direction. In these cases, the vector sum, and thus $M_{T}^{R}$, will have a large value. $M_{\text{T}}^{R}$ is another measure of the scale of the event that only uses transverse quantities in contrast to longitudinal quantities in $M_{R}'$.

Finally a razor variable is defined to discriminate between signal and background:

4 $$ R = \frac{ M_{\text{T}}^R }{ M_{R}' } . $$

This variable takes low values for multijet-like events and tends to be uniformly distributed between 0 and 1 for sparticle decay-like events, providing good discrimination against backgrounds without genuine $E_{\mathrm{T}}^{\mathrm{miss}}$. The impact of some important experimental uncertainties, like the jet energy scale uncertainty, are reduced in this ratio. In an analysis based on the razor variables, a cut on *R* can be used to eliminate these backgrounds before a SUSY search is made in the distribution of the variable $M_{R}'$.

## Data and Monte Carlo samples

The data included in this analysis were collected between March and October 2011. After basic trigger and data quality requirements, the full dataset corresponds to 4.7±0.2 fb^−1^ [27, 28].

Events in the zero-lepton channels are selected using a trigger that requires a jet with transverse momentum *p*_T_>100 GeV at L1. In the event filter, *H*_T_>400 GeV is required, where *H*_T_ is calculated through a scalar sum of the *p*_T_ of all calorimeter objects with *p*_T_>30 GeV and |*η*|<3.2. With the exception of a cross-check of the multijet background estimate, which uses prescaled single-jet triggers, this trigger requirement is fully efficient for the offline selection used in the analysis.

The one- and two-lepton channels make use of the lowest-*p*_T_ single-lepton triggers available for the entire running period. The muon triggers require a muon with *p*_T_>18 GeV, and the electron triggers require an electron with *p*_T_>22 GeV. Offline, the leading lepton in the event is required to have *p*_T_>20 GeV (*p*_T_>25 GeV) if it is a muon (electron), in order to ensure that the triggers are fully efficient with respect to the offline event selection. For the two-lepton analysis, where there are overlaps in the triggers, the electron trigger takes priority over the muon trigger.

Offline, an event is required to have at least one vertex with at least five tracks associated to it, each with $p_{\mathrm{T}} ^{\rm track}>400~\mbox{MeV}$. This requirement reduces cosmic ray and beam-related backgrounds. The primary vertex is defined as the one with the largest $\sum( p_{\mathrm{T}} ^{\rm track})^{2}$ of the associated tracks. Events that suffer from sporadic calorimeter noise bursts or data integrity errors are also rejected.

Monte Carlo (MC) simulated events were used to develop the analysis and assist in estimations of background rates. All MC samples are processed through ATLAS’s full detector simulation [29] based on Geant4 [30], which was run with four different configurations corresponding to detector conditions of four distinct operating periods of 2011. The fractions of MC simulation events in these four periods match the fractions of data in each period. During the data collection, the average number of proton–proton collisions per bunch crossing in addition to the one of interest (“event pile-up” or simply “pile-up”) increased from approximately two to twelve. To mimic the effect of pile-up, additional inelastic proton–proton collisions are generated using Pythia [31] and overlaid on top of every MC event. Within each period, the profile of the average number of events per bunch crossing (〈*μ*〉) is re-weighted to match the data in that period. The same trigger selection is applied to the MC simulation events, which are then passed through the same analysis code as the data. Reconstruction and trigger efficiency scale factors are applied to the MC simulation in order to take into account small discrepancies between the data and the MC simulation.

Table 1 lists the major backgrounds along with the chosen estimation method (described in Sect. 7) and the primary and alternative MC generators used in this analysis. In all cases, MC@NLO and Alpgen are interfaced to Herwig and Jimmy for the parton shower, hadronisation, and underlying event modelling. The multijet background is normalised to the leading order generator cross-section predicted by Pythia. The $t\bar{t}$ production cross-section of 166.8 pb is calculated at approximate NNLO in QCD using Hathor [32] with the MSTW2008 NNLO PDF sets [33]. The calculation is cross-checked with an NLO+NNLL calculation [34] implemented in Top++ [35]. The single-top production cross-sections are calculated separately for *s*-channel, *t*-channel, and *Wt* production at NNLO [36–38].

**Table 1:** Background estimation methods, primary and alternative MC event generators, and normalisation uncertainties for each of the major backgrounds. The backgrounds are constrained using various Control Regions (CRs) that are enriched in certain samples (see Sect. 6). The $t\bar{t}$ background estimate includes small contributions from $t\bar{t} W$ and $t\bar{t} Z$, generated with MadGraph. The diboson *WW* background estimate also includes *W* ^±^ *W* ^±^ *jj* generated with MadGraph. The last column of the table indicates the uncertainty on the normalization in the simultaneous fit used to test signal hypotheses. “None” indicates that the normalization is fully constrained in the fit. The grouping indicates the samples that are combined and jointly varied in the fit. Within a group, the relative normalizations are fixed

Background	0-Lepton	1-Lepton	2-Lepton	Generator	Alternate	Normalisation uncertainty
Multijets	MJ CRs	Matrix method	Matrix method	Pythia [31]	Alpgen [43]	None
*W*→*ℓν*	*W* CRs	*W* CRs	Matrix method	Alpgen [43]		None (grouped with *Z*)
*Z*→*ℓℓ*	*Z* CRs	*Z* CRs	*Z* CRs	Alpgen [43]		None (grouped with *W*)
Drell–Yan	*Z* CRs	*Z* CRs	*Z* CRs	Alpgen [43]		None (grouped with *W*)
*Z*→*νν*	*Z* CRs	Matrix method	Matrix method	Alpgen [43]		None (grouped with *W*/*Z*)
$t\bar{t}$(had)	$t\bar{t}$ CRs	$t\bar{t}$ CRs	$t\bar{t}$ CRs	MC@NLO [44]		None
$t\bar{t}$(leptonic)	$t\bar{t}$ CRs	$t\bar{t}$ CRs	$t\bar{t}$ CRs	Alpgen [43]	MC@NLO [44–47]	None
Single top	$t\bar{t}$ CRs	$t\bar{t}$ CRs	$t\bar{t}$ CRs	MC@NLO [44]		None (grouped with $t\bar{t}$)
*WW* diboson	MC	MC	MC	Herwig [48]	Alpgen [43]	NLO ± 30 %
Other diboson	*Z* CRs	*Z* CRs	*Z* CRs	Herwig [48]	Alpgen [43]	None (grouped with *W*/*Z*)

The *W* and *Z* (including Drell–Yan with *m*_*ℓℓ*_>40 GeV) production cross-sections of 10.46 nb and 0.964 nb are calculated at NNLO using FEWZ [39]. For the production of vector bosons in association with heavy flavour, in accordance with ATLAS measurements [40], the production cross-section for $W+ \bar{b} $ and $W+ c\bar{c} $ are scaled by 1.63, and the cross-section for *W*+*c* is scaled by 1.11 compared to the NLO cross-section [41]. Additional uncertainties on the production of *W* and *Z* bosons in association with heavy flavour of 45 % for $W+ \bar{b} $ and $W+ c\bar{c} $, 32 % for *W*+*c*, and 55 % for $Z+b \bar{b} $ are included. Alpgen describes the jet multiplicity and inclusive $M_{R}'$ distributions well, but it does not correctly model the vector boson *p*_T_ distribution. Therefore, the boson *p*_T_ in the Alpgen samples is re-weighted according to the distribution produced Sherpa. Half of the difference between the weight and unity is applied as a systematic uncertainty on the re-weighting procedure. Further systematic uncertainties on the shapes of Alpgen samples are derived by systematically varying the generator parameters, including matching and factorisation scales. Diboson production cross-sections of 44.92 pb, 17.97 pb, and 9.23 pb for *WW*, *WZ*, and *ZZ* (including off-shell production with *m*_*ℓℓ*_>12 GeV) are calculated at NLO using MCFM [42]. In order to avoid low-mass resonances, all dilepton events are required to have the invariant mass *m*_*ℓℓ*_>20 GeV. These cross-sections provide the starting normalisations for all background processes.

Two SUSY-inspired simplified models are used for the interpretation of the results from this search. The first considers gluino pair-production, with the gluino decaying to a $t\bar{t}$ pair and the LSP via an off-shell stop. This model is generated using Herwig++ [49], with the gluino and LSP masses being the only free parameters. The top quarks are required to be on-shell, limiting the mass splitting between the gluino and the LSP to greater than 2×*m*_top_.

The second considers gluino pair-production, with the gluino decaying to two quarks and a chargino via an off-shell squark. The chargino then decays to a *W* boson and the LSP. The free parameters of this model are the masses of the gluino, chargino, and LSP. For convenience, two two-dimensional planes are generated: one with the chargino mass exactly between the masses of the gluino and the LSP and one with the mass of the LSP fixed to 60 GeV. Because initial-state radiation can be important for the acceptance of these models when the mass splitting between the gluino and LSP is small, this model is generated using MadGraph [50] with at most one additional jet in the matrix element. Pythia is used for the parton shower and hadronisation. Systematic uncertainties on matrix element matching and initial-state radiation modelling are included, leading to 20 % uncertainties for small mass splittings and small gluino masses, but no uncertainty for mass splittings above 200 GeV and masses above 400 GeV.

Additionally, the results are interpreted in terms of SUSY signal models based on the constrained minimal supersymmetric model (CMSSM or MSUGRA) [18–22]. The parameters of this model are the high-energy-scale universal scalar mass, *m*_0_, the universal gaugino mass, *m*_1/2_, the ratio of the vacuum expectation values of the two Higgs fields, tan(*β*), the tri-linear coupling strength, *A*_0_, and the sign of the Higgsino mass parameter, *μ*. Samples are generated in a two-dimensional grid of the *m*_0_–*m*_1/2_ parameters where tan(*β*)=10 and *A*_0_=0 are fixed and *μ* is set positive. This MC data grid is generated using Herwig++ [49], with a more dense population of points at low mass. IsaSUSY [51] is used to run the high-energy-scale parameters down to the weak-scale.

Signal cross-sections are calculated to next-to-leading order in the strong coupling constant, adding the resummation of soft gluon emission at next-to-leading-logarithmic accuracy (NLO+NLL) [52–56]. The nominal cross-section and the uncertainty are taken from an envelope of cross-section predictions using different PDF sets and factorisation and renormalisation scales, as described in Ref. [57]. For each of these signal models, the luminosity systematic uncertainty of 3.9 % [27, 28] and statistical uncertainty, typically of order 10 %, is included.

## Physics object identification and selection

Events are categorised into six exclusive samples defined by the presence of zero, one, or two leptons, with or without *b*-tagged jets. The particle candidate selections that define these samples are referred to as the “baseline” object selection. Since a particle may simultaneously satisfy multiple particle hypotheses (e.g. electron and jet), an overlap removal procedure (described below) assigns a unique interpretation to each candidate. The selections are then refined to enhance signal candidates whilst removing leptons not originating from gauge bosons, tau-leptons or sparticles.

Baseline electrons are required to have *E*_T_>10 GeV, be within the fiducial acceptance of the inner detector (|*η*|<2.47), and pass a version of the “medium” selection criteria [58] updated for 2011 running conditions, which requires hadronic calorimeter energy deposition and a calorimetric shower shape consistent with an electron and a match to a good quality inner detector track. Signal electrons are required to be isolated from other objects and satisfy “tight” selections. The tight selection applies stricter track quality and matching than medium and ensures the number of hits in the transition radiation tracker is consistent with the electron hypothesis. The isolation requirement is that the sum of the *p*_T_ of all charged particle tracks associated with the primary vertex within Δ*R*=0.2, where $\Delta R = \sqrt{(\Delta\eta)^{2} + (\Delta\phi)^{2}}$, of the electron is less than 10 % of the electron *E*_T_. In the leptonic channels, if the leading lepton in a data event is an electron, it is additionally required to match an EF trigger electron. MC simulation events are re-weighted to compensate for mis-modelling of the single-lepton trigger efficiency. The energy of electrons in simulated events is also smeared prior to object selection in order to reproduce the resolution in *Z* and *J*/*ψ* data. Finally, in order to account for percent-level differences in electron reconstruction efficiency, *η*- and *E*_T_-dependent scale factors, derived from *Z*, *W*, and *J*/*ψ* events in the data, are applied to each simulated electron satisfying overlap removal and selection requirements.

Baseline muons are reconstructed as either a combined track in the muon spectrometer and inner detector, or as an inner detector track matching with a muon spectrometer segment [59]. Tracks are required to have good quality, and the muon is required to have *p*_T_>10 GeV and |*η*|<2.4. Signal muons are required to be isolated by ensuring that the sum of the *p*_T_ of all charged particle tracks associated with the primary vertex within Δ*R*=0.2 of the muon is less than 1.8 GeV. Matching to EF trigger muons in data, MC event trigger re-weighting, muon momentum smearing, and MC/data efficiency scaling are performed in a similar way for muons as electrons (described above) [60–62]. These corrections are typically percent or sub-percent level.

Calorimeter jets are reconstructed from topological clusters of energy deposited in the calorimeter calibrated at the electromagnetic (EM) scale [63] using the anti-*k*_*t*_ jet algorithm [64, 65] with a four-momentum recombination scheme and a distance parameter of 0.4. Jets reconstructed with an EM-scale *p*_T_>7 GeV are calibrated to the hadronic scale (particle level) using *p*_T_ and *η*-dependent factors, derived from simulation and validated with test beam and collision data [66]. In order to remove specific non-collision backgrounds, events are rejected if they contain a reconstructed jet that does not pass several quality and selection criteria [66]. Signal jets are selected if they lie within |*η*|<2.5 with a jet vertex fraction (JVF) of at least 75 %, where the JVF is the fraction of summed *p*_T_ of the tracks associated with the jet that is carried by tracks consistent with the primary vertex of the event, thus associating the jet with the *pp* collision of interest. Jets are tagged as heavy flavour using the combined neural network “jet fitter” algorithm [67] with the 60 % efficiency working point. Scale factors for heavy flavour jets are used in MC simulation in order to reproduce the expected *b*-jet identification performance in data.

In order to ensure that objects are not double counted, overlaps between objects are removed using a hierachical procedure. If any two baseline electrons lie within a distance of Δ*R*=0.1 of one another, the electron with the lower calorimeter *E*_T_ is discarded. Next, jets passing basic selections are required to be at least 0.2 units away from all surviving baseline electrons in *η*–*ϕ*. Electrons are then required to be at least 0.4 units away from surviving jets. Finally, in order to mitigate the effect of jets which have deposited significant energy in the muon spectrometer on mass measurements and reduce the number of events with badly measured missing transverse momentum, muons with *p*_T_>250 GeV within Δ*R*=0.2 of a jet with *p*_T_>500 GeV are removed. A negligible number of events in the data are removed by this cut.

Following these overlap removal procedures, the missing transverse momentum and razor variables are calculated. The determination of the missing transverse momentum uses all baseline electrons with *E*_T_>20 GeV, all baseline muons, all calibrated jets with *p*_T_>20 GeV, and EM scale topological calorimeter clusters not belonging to any object. Note that in the MC simulation, objects enter this calculation after the energy or *p*_T_ smearing described above.

In counting leptons for event classification, baseline electrons and muons are then required to be at least 0.4 units away from all good jets in *η*–*ϕ*. If an electron and muon are separated by Δ*R*_cone_<0.1, neither is counted.

In order to remove events with large missing transverse momentum due to cosmic rays, events are vetoed if they contain a muon in which the transverse and longitudinal impact track parameters are greater than 0.2 mm and 0.1 mm with respect to the primary vertex, respectively. The vertex resolution is significantly smaller than either of these requirements, typically <0.05 mm. Also vetoed are events with badly measured, non-isolated muons. These muons with large momentum uncertainties are rare in both the signal and background events and can have significant impact on the $E_{\mathrm{T}}^{\mathrm{miss}}$ and razor variables.

During a portion of the run period, a hardware failure resulted in a region of the calorimeter not being read out. For data collected during this period, and for a corresponding fraction of the MC samples, events are rejected if they fail the “smart LAr hole veto” [13]. This ensures that if an event contains one or more jets pointing to the dead region and those jets may contribute substantially to the missing transverse momentum in the event, the event is discarded.

Signal regions are defined after all overlap removal is complete. Events with no baseline leptons and events with the highest-*p*_T_ lepton below the leading lepton requirement (25 GeV for electrons, 20 GeV for muons) are accepted into the zero-lepton regions. Events with one leading lepton satisfying all requirements, including that on leading lepton *p*_T_ (above), and no other baseline leptons with *p*_T_>10 GeV are accepted into the one-lepton regions. Events with exactly one additional signal lepton above 10 GeV and no other baseline leptons are accepted into the two-lepton regions.

## Search technique

**Fig. 1:**
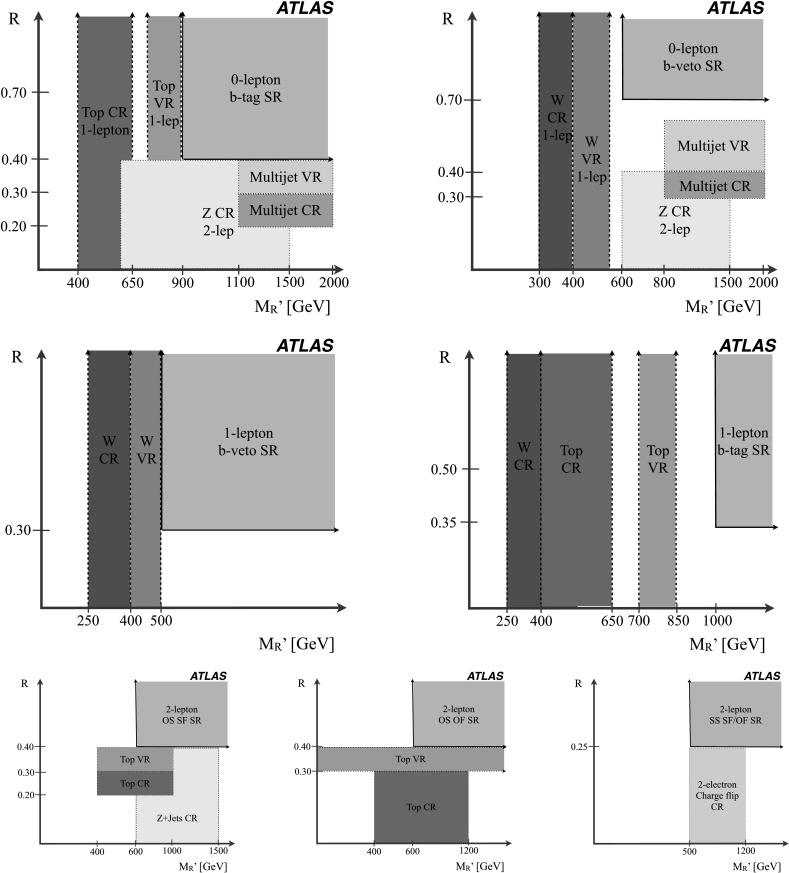
A visual representation of the zero-lepton (*top*), one-lepton (*middle*), and two-lepton (*bottom*) control validation (VR), and signal (SR) regions. The CR and VR regions also indicate the respective dominant background. Regions with two leptons are classified as same-sign (SS) or opposite-sign (OS) events and as same-flavor (SF) or opposite-flavor (OF) events

After sorting events into the six samples described in the previous section, each sample is further divided in the *R*–$M_{R}'$ plane into control regions (CR), which are choosen so that they are dominated by a specific background, and signal regions (SR). Additionally, validation regions (VR) are constructed, which do not constrain the background but are used to evaluate the agreement between data and MC simulation. Table 2 lists these regions, which are also visualised in the *R*–$M_{R}'$ plane in Fig. 1. These regions are binned in either *R* or $M_{R}'$ and then simultaneously fit to MC estimates for background and signal rates with correlations from sample to sample and region to region taken into account. The hadronic (had.) and one-lepton signal regions are divided into events with and without *b*-tagged jets (“b-tag” and “b-veto,” respectively). The two-lepton events are divided into regions with opposite-sign (OS) and same-sign (SS) leptons and regions with opposite-flavour (OF) and same-flavour (SF) leptons. While some background components are sufficiently constrained by the CRs to be left free in the fit, others are constrained to estimates derived from other techniques or MC simulation. Table 1 summarises the backgrounds, the estimation technique, the source of the estimate, and the normalization uncertainty used in the fit. Finally, systematic uncertainties on all backgrounds are included as nuisance parameters. The result is a maximum likelihood fit that encapsulates all knowledge about the background and signal consistently across all channels.

**Table 2:** Background control, signal, and validation regions. All signal regions include the overflow in the highest bin. “N/A” means that there is no requirement. Regions with two leptons are classified as same-sign (SS) or opposite-sign (OS) events and as same-flavor (SF) or opposite-flavor (OF) events. The binning of the validation regions does not affect the results, so they are listed as “N/A”

Name	Leptons	*b*-jets	*N*_Jets_	*R* range	$M_{R}'$ range	Number of bins
Control regions						
Had. *b*-veto Multijet	0 leptons	=0	>5	0.3<*R*<0.4	$800< M_{R}'<2000~\mbox{GeV}$	12 in $M_{R}'$
Had. *b*-tag Multijet	0 leptons	>0	>5	0.2<*R*<0.3	$1000<M_{R}'<2000~\mbox{GeV}$	10 in $M_{R}'$
*e* *W*+jets	1 electron	=0	>5	0<*R*<0.7	$300< M_{R}'<400~\mbox{GeV}$	7 in *R*
*μ* *W*+jets	1 muon	=0	>5	0<*R*<0.7	$300< M_{R}'<400~\mbox{GeV}$	7 in *R*
*e* $t\bar{t}$	1 electron	>0	>5	0<*R*<0.7	$400< M_{R}'<650~\mbox{GeV}$	7 in *R*
*μ* $t\bar{t}$	1 muon	>0	>5	0<*R*<0.7	$400< M_{R}'<650~\mbox{GeV}$	7 in *R*
*ee* $t\bar{t}$	2 OS electrons	>0	N/A	0.2<*R*<0.3	$400< M_{R}'<1000~\mbox{GeV}$	6 in $M_{R}'$
*μμ* $t\bar{t}$	2 OS muons	>0	N/A	0.2<*R*<0.3	$400< M_{R}'<1000~\mbox{GeV}$	6 in $M_{R}'$
*eμ* $t\bar{t}$	2 OS OF leptons	>0	N/A	*R*<0.3	$400< M_{R}'<1200~\mbox{GeV}$	8 in $M_{R}'$
*ee* *Z*	2 OS electrons	N/A	N/A	*R*<0.4	$600< M_{R}'<1500~\mbox{GeV}$	9 in $M_{R}'$
*μμ* *Z*	2 OS muons	N/A	N/A	*R*<0.4	$600< M_{R}'<1500~\mbox{GeV}$	9 in $M_{R}'$
*ee* Charge flip	2 SS electrons	N/A	N/A	*R*<0.25	$500< M_{R}'<1200~\mbox{GeV}$	7 in $M_{R}'$
Signal regions						
Had. *b*-veto	0 leptons	=0	>5	*R*>0.70	$600< M_{R}'<1200~\mbox{GeV}$	3 in $M_{R}'$
Had. *b*-tag	0 leptons	>0	>5	*R*>0.40	$900< M_{R}'<1500~\mbox{GeV}$	3 in $M_{R}'$
*e* *b*-veto	1 electron	=0	>5	*R*>0.55	$500< M_{R}'<1000~\mbox{GeV}$	3 in $M_{R}'$
*e* *b*-tag	1 electron	>0	>5	*R*>0.35	$1000< M_{R}'<1600~\mbox{GeV}$	6 in $M_{R}'$
*μ* *b*-veto	1 muon	=0	>5	*R*>0.55	$500< M_{R}'<1000~\mbox{GeV}$	3 in $M_{R}'$
*μ* *b*-tag	1 muon	>0	>5	*R*>0.35	$1000< M_{R}'<1400~\mbox{GeV}$	4 in $M_{R}'$
OS-*ee*	2 OS electrons	N/A	N/A	*R*>0.40	$600< M_{R}'<1000~\mbox{GeV}$	4 in $M_{R}'$
OS-*μμ*	2 OS muons	N/A	N/A	*R*>0.40	$600< M_{R}'<1000~\mbox{GeV}$	4 in $M_{R}'$
SS-*ee*	2 SS electrons	N/A	N/A	*R*>0.25	$500< M_{R}'<900~\mbox{GeV}$	4 in $M_{R}'$
SS-*μμ*	2 SS muons	N/A	N/A	*R*>0.25	$500< M_{R}'<900~\mbox{GeV}$	4 in $M_{R}'$
OS-*eμ*	2 OS OF leptons	N/A	N/A	*R*>0.40	$600< M_{R}'<1000~\mbox{GeV}$	4 in $M_{R}'$
SS-*eμ*	2 SS OF leptons	N/A	N/A	*R*>0.25	$500< M_{R}'<900~\mbox{GeV}$	4 in $M_{R}'$
Validation regions						
Had. *b*-veto Multijet	0 leptons	=0	>5	0.4<*R*<0.6	$800< M_{R}'<2000~\mbox{GeV}$	N/A
Had. *b*-tag Multijet	0 leptons	>0	>5	0.3<*R*<0.4	$1100<M_{R}'<2000~\mbox{GeV}$	N/A
1-lep *b*-veto *W*+jets	1 lepton	=0	>5	N/A	$400< M_{R}'<550~\mbox{GeV}$	N/A
1-lep *b*-tag $t\bar{t}$	1 lepton	>0	>5	N/A	$700< M_{R}'<850~\mbox{GeV}$	N/A
OS-*ee*/*μμ* $t\bar{t}$	2 OS SF leptons	>0	N/A	0.3<*R*<0.4	$400~\mbox{GeV}< M_{R}'$	N/A
OS-*eμ* $t\bar{t}$	2 OS OF leptons	>0	N/A	0.3<*R*<0.4	N/A	N/A

When evaluating a signal hypothesis, any signal contamination in the control regions is taken into account for each signal point, as the control region fits are performed for each signal hypothesis. Separately, each signal region (one at a time), along with all control regions, is also fit under the background-only hypothesis. This fit is used to characterise agreement in each signal region with the background-only hypothesis and to extract visible cross-section limits and upper limits on the production of events from new physics (*N*_BSM_).

The fit considers several independent background components:

- $t\bar{t}$ *and single top*. A total of five top control regions are defined in the one- and two-lepton channels. The normalisation of this component is allowed to vary freely in the fit.
- *Bosons, except diboson* *WW*. The inclusion of the *WZ* and *ZZ* diboson samples is motivated by the dominance of leptonic *Z* decays in the two-lepton signal regions, the dominance of *Z*→*νν* in the zero-lepton signal regions, and the dominance of $WZ\rightarrow\ell\nu q\bar{q} $ in the one-lepton signal regions. In all of these cases, the experimental uncertainties affect the samples in the same way as they do *W*+jets or *Z*+jets, and therefore they are combined in order to treat them as fully correlated. The normalisation of this sample is allowed to vary freely in the fit. Independent validation of the *Z*+jets background is carried out in two-lepton control regions. The agreement is good between data and MC simulation in both normalisation and shape.
- *Diboson* *WW*. This sample is constrained with a 30 % cross-section systematic uncertainty. The constraint is necessary because of the relatively small contribution of the sample in most signal and control regions and because no *WW*-dominated control region can be constructed; if the background were allowed to vary freely, then the fit may find a minimum with an unreasonably large or small contribution from diboson *WW* events and hide some other effect with an artificial *WW* normalisation.
- *Charge flip*. Charge mis-identification can occur due to physical effects, like lepton Bremsstrahlung, and detector effects, especially for high-*p*_T_ leptons with almost straight tracks. These effects generate background in the same-sign dielectron and electron-muon channels. This background is negligible in the dimuon channel, where the contribution from both physical and detector effects is far smaller. The electron charge-flip rate is measured as a function of *η* in the data [68], allowing MC simulation to model the lesser dependence on *p*_T_. These charge-flip rates are applied to opposite-sign MC simulation events, providing an estimate of the overall contribution from charge flip in these channels. The electron *p*_T_ is additionally shifted and smeared to mimic the effect of charge mis-identification. This shift in the *p*_T_ is propagated through to the razor variables. The uncertainty from the charge flip probabilities dominates the uncertainty of this background.
- *Fake leptons*. The multijet background in the one-lepton signal regions, as well as the *W*+jets, semi-leptonic $t\bar{t}$, and multijet background in the two-lepton signal regions, comes predominantly from hadrons faking electrons and muons. This background is estimated using the “matrix method” [13, 68], using the number of baseline leptons not passing signal lepton requirements. The efficiency for a real lepton passing the baseline lepton requirements to pass the signal lepton requirements is estimated using *Z* MC simulation events. The rejection rate for fake leptons is estimated in data, using samples enriched in fake leptons. For electrons, the factors are derived and applied separately for inclusive samples of events and samples requiring a *b*-tagged jet. Because this background accounts for *all* fake background, MC events in the one-lepton (two-lepton) channels are required to have at least one (two) prompt lepton(s) from a *τ* lepton, *W* boson, *Z* boson, or sparticle. The uncertainty on this background estimate has a statistical component from the number of events in the control region and a systematic component from the uncertainty on the scale factors.
  Some fraction of the events with same-sign, baseline leptons in the data may be due to charge flip. Thus, the matrix method overestimates somewhat the fake lepton background in the dilepton channels. In order to correct for this overlap, opposite-sign events in data containing baseline leptons that do not pass the signal lepton requirements are used. Each event is assigned a weight representing the likelihood of that event being subject to charge mis-identification. The weighted events are then presented as a negative component to the same-sign fake background distribution, such that the contribution to the same sign fake background from originally oppositely charged leptons is subtracted.
- *Multijets in zero-lepton channel.* Two specific control regions constrain this background, and its normalisation is allowed to vary freely in the fit. Several different approaches are used to cross-check this estimate. Prescaled single jet triggers are used to construct independent multijet-enriched control regions at low $M_{R}'$ that is free from the inefficiency of the *H*_T_-based trigger. The observed number of events in this region are then projected into the signal region using transfer factors from MC simulation. Alternatively, in order to model the mis-measurement of jets in the calorimeter, jets in events collected with these single-jet triggers are smeared according to response functions estimated using data [10]. Both of these methods result in an estimate consistent with that derived in the main fit.

The systematic effects included as nuisance parameters in the fit are: the jet energy scale and resolution uncertainties; *b*-tagging uncertainties; uncertainty on the MC simulation modelling of the JVF; the additional cross-section uncertainty on the production of heavy flavour in association with a vector boson; the uncertainties on trigger efficiency and matching and reconstruction efficiency; a systematic uncertainty on the re-weighting of the *W*-boson *p*_T_; uncertainties on the missing transverse momentum pile-up dependence and the calibration of energy not associated with an object in the event; the matrix method statistical and systematic uncertainties; the charge flip systematic uncertainties; the diboson *WW* shape systematic uncertainty taken from comparing Herwig to Alpgen. Where the systematic uncertainties affect object definitions, corrections are propagated to the missing transverse momentum and razor variable calculations. The effects of other uncertainties on the final results are negligible. These uncertainties affect the signal yield and shape in the signal regions, as well as the allowed variation in signal-region background estimates after the control region constraints. In most signal regions, the jet energy scale uncertainty is the dominant experimental uncertainty (from 10 % to 25 %).

## Background fit

**Fig. 2:**
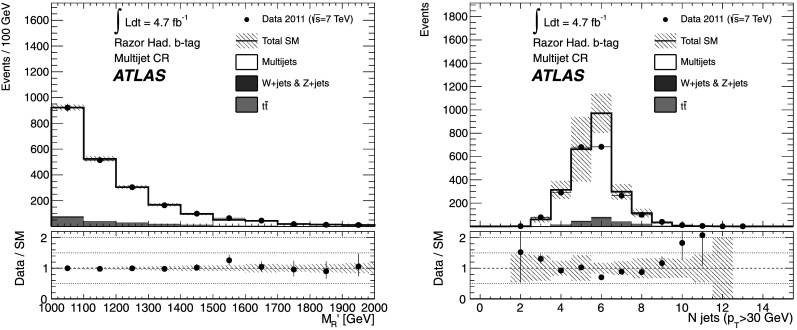
The distribution of $M_{R}'$ (*left*) and the number of jets with *p*_T_>30 GeV (*right*) in the multijet control region with a *b*-tagged jet requirement (*dots* with error bars), the expectation from the control region fit for various backgrounds (*filled*), and the systematic uncertainty (*hatched*)

Figure 2 shows the distributions of $M_{R}'$ and jet multiplicity in the zero-lepton multijet control region with a *b*-tagged jet requirement, with results from the fit to the control regions overlayed. By design, the multijet background is dominant in these regions. The small contribution from $t\bar{t}$ and *W*+jets backgrounds are constrained by other control regions in the simultaneous fit. The hatched area indicates the total systematic uncertainty after the constraints imposed by the fit.

**Fig. 3:**
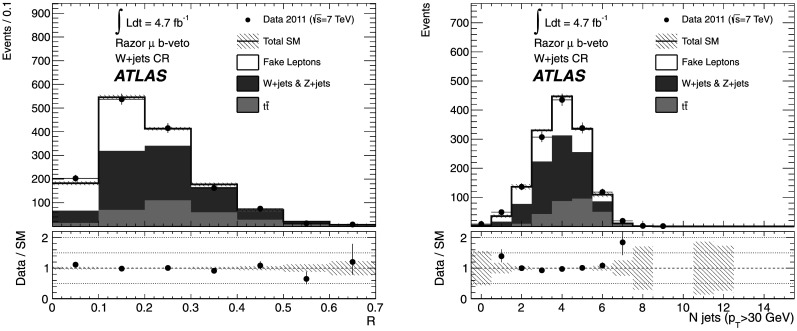
The distribution of *R* (*left*) and the number of jets with *p*_T_>30 GeV (*right*) in the *W*→*μν*+jets control region (*dots* with error bars), the expectation from the control region fit for various backgrounds (*filled*), and the systematic uncertainty (*hatched*). Error bands on the ratios are only shown for bins with non-zero MC simulation predictions. In the high jet-multiplicity bins, the MC simulation statistics are poor

The distributions of *R* and jet multiplicty for the backgrounds after the control region fit in the *W*→*μν*+jets control region are shown in Fig. 3. The control region at low *R* is dominated by fake backgrounds, and at moderate-to-high *R* they are dominated by *W*+jets. The use of an alternate control region with a cut on transverse mass, which significantly reduces the fake contribution, results in a negligible change in the final search results.

**Fig. 4:**
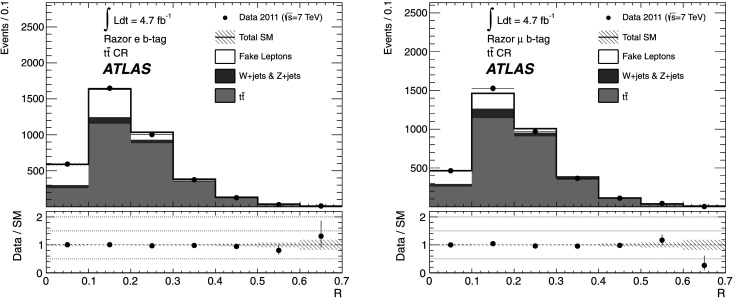
The distribution of *R* in the one-lepton $t\bar{t}$ control regions (*dots* with error bars), the expectation after the control region fit for various backgrounds (*filled*), and the systematic uncertainty (*hatched*)

**Fig. 5:**
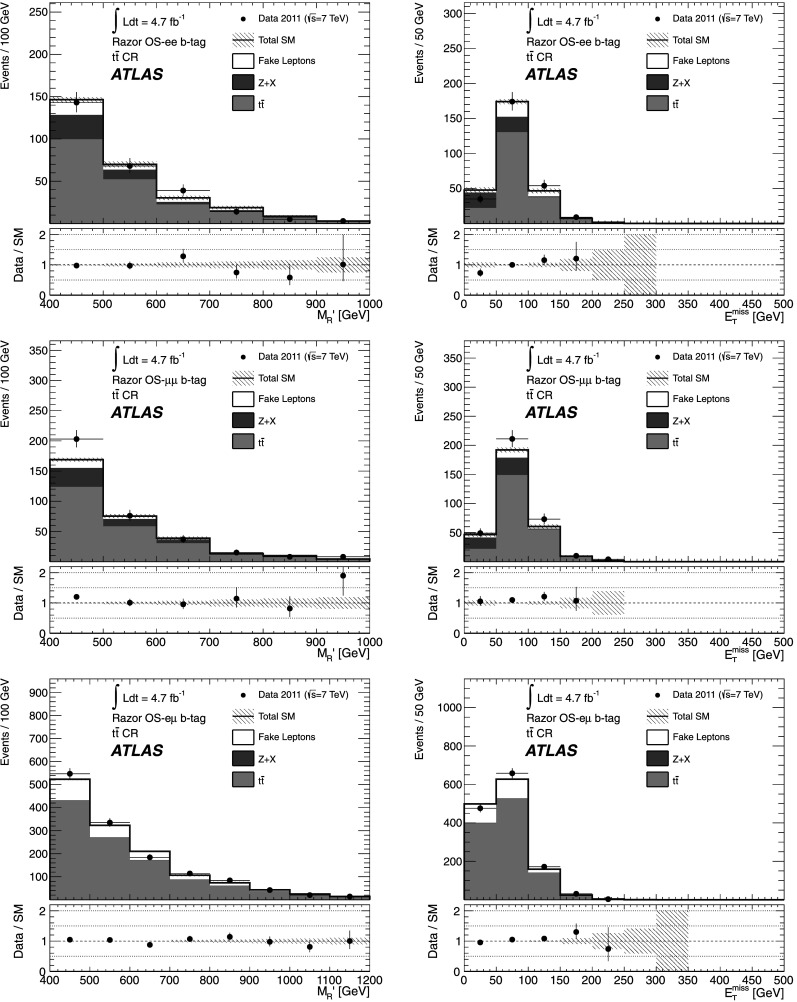
The distribution of $M_{R}'$ (*left*) and missing transverse momentum (*right*) in the two-lepton $t\bar{t}$ control regions (*dots* with error bars), the expectation after the control region fit for various backgrounds (*filled*), and the systematic uncertainty (*hatched*)

Figures 4 and 5 show the one-lepton and two-lepton $t\bar{t}$ control regions, respectively. The fit reduces the normalisation of the $t\bar{t}$ background in the one-lepton control region by approximately 15–20 % with respect to the unmodified expectation from MC simulation. This shift predominantly affects the semi-leptonic $t\bar{t}$ background. In the two-lepton analysis, there is a significant contribution to the background expectation from *Z*-boson events with heavy flavour, particularly at low $E_{\mathrm{T}}^{\mathrm{miss}}$. The lowest $M_{R}'$ bin shows the most significant disagreement, which demonstrates the importance of shape profiling by binning the control regions. Although in that lowest bin, particularly in the two-muon channel, the MC simulation underestimates the amount of data, a single-binned normalisation of the $t\bar{t}$ background would result in an overestimation of the background at high $M_{R}'$. The distributions of missing transverse momentum are also shown for the two-lepton control regions.

**Fig. 6:**
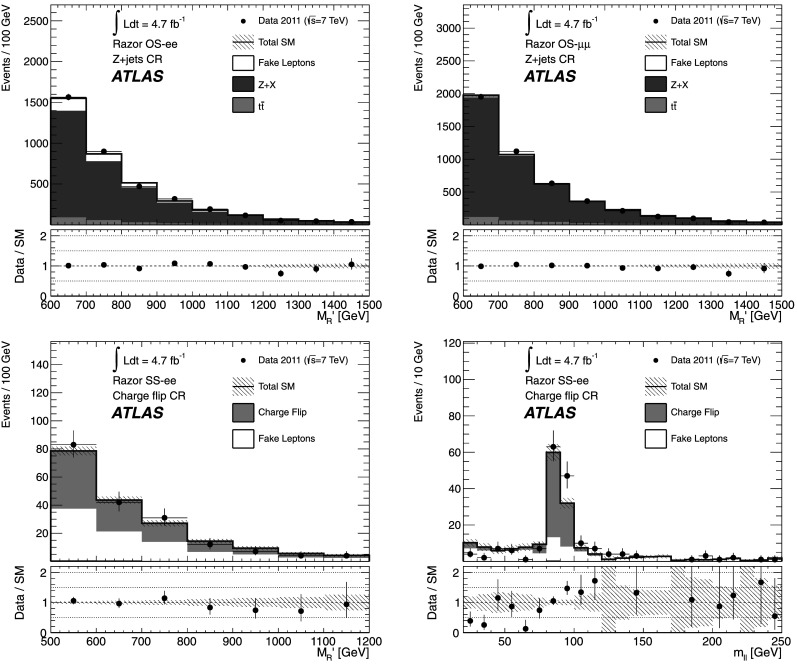
Top, the distribution of $M_{R}'$ in the *Z*+jets control regions. Bottom, the distribution of $M_{R}'$ (*left*) and dilepton mass (*right*) in the charge flip control region (*dots* with error bars), the expectation after the control region fit for various backgrounds (*filled*), and the systematic uncertainty (*hatched*)

The distributions of $M_{R}'$ for the two *Z*+jets control regions are shown in Fig. 6. After the control region fit, good agreement is observed in both the electron and muon channels.

The charge flip background is significant in the same-sign two-electron channel. Figure 6 also shows the distribution of $M_{R}'$ in a charge flip enriched control region. There remain significant uncertainties on the background even after the control region fit, since it is dominated by charge flip and fake leptons, both of which have large systematic uncertainties associated with them. The distribution of dilepton mass is also shown.

The contributions to each of the control regions before and after the fit to the control regions are shown in Tables 3 and 4.

**Table 3:** The number of observed events and the results of the background-only fit to the control regions in the zero- and one-lepton control regions, for an integrated luminosity of 4.7 fb^−1^. Nominal MC expectations (normalised to MC cross-sections) are given for comparison. The errors shown are the statistical plus systematic uncertainties

Control region	Had. *b*-veto Multijet	Had. *b*-tag Multijet	*e* *W*+jets	*μ* *W*+jets	e $t\bar{t}$	*μ* $t\bar{t}$
Observed events	1032	2153	1833	1413	3783	3479
Fitted background events	1030±30	2150±50	1840±40	1410±30	3820±60	3470±50
	Fitted background decomposition					
Fitted top events	21±7	170±19	280±30	290±30	2800±60	2800±60
Fitted *W*/*Z* events	90±10	26±4	670±40	690±50	210±20	240±30
Fitted *WW* diboson events	0.54±0.18	0.14±0.05	4.2±1.8	4.5±1.9	1.2±0.5	1.0±0.4
Fitted multijet events	920±30	1960±50	0±0	0±0	0±0	0±0
Fitted charge flip events	0±0	0±0	0±0	0±0	0±0	0±0
Fitted fake lepton events	0±0	0±0	890±50	430±40	810±70	440±60
Expected background events	990	2670	2110	1560	4300	3790
	Expected background decomposition					
MC exp. top events	52	245	450	470	3300	3250
MC exp. *W*/*Z* events	110	28	740	760	200	220
MC exp. *WW* diboson events	0.61	0.18	4.6	4.6	1.4	1.1
MC exp. multijet events	830	2400	0	0	0	0
Charge flip events (estimated from data)	0	0	0	0	0	0
Fake lepton events (estimated from data)	0	0	910	330	800	310

**Table 4:** The number of observed events and the results of the background-only fit to the control regions in the two-lepton control regions, for an integrated luminosity of 4.7 fb^−1^. Nominal MC expectations (normalised to MC cross-sections) are given for comparison. The errors shown are the statistical plus systematic uncertainties

Control region	ee $t\bar{t}$	*μμ* $t\bar{t}$	*eμ* $t\bar{t}$	*ee* *Z*	*μμ* *Z*	ee Charge flip
Observed events	272	347	1340	3688	4579	183
Fitted background events	277±14	310±10	1320±30	3670±60	4590±70	183±13
	Fitted background decomposition					
Fitted top events	198±7	237±8	1090±30	220±9	281±11	0.104±0.011
Fitted *W*/*Z* events	45±4	51±5	3.5±0.3	3090±90	4220±80	1.06±0.11
Fitted *WW* diboson events	0.22±0.08	0.10±0.15	1.3±0.5	6±3	8±5	1.2±0.6
Fitted multijet events	0±0	0±0	0±0	0±0	0±0	0±0
Fitted charge flip events	0±0	0±0	0±0	0±0	0±0	94±14
Fitted fake lepton events	34±15	22±8	220±40	360±100	80±50	87±19
Expected background events	305	336	1340	3920	5050	148
	Expected background decomposition					
MC exp. top events	225	276	1220	278	357	0.094
MC exp. *W*/*Z* events	41	47	3.1	3360	4600	1.14
MC exp. *WW* diboson events	0.21	0.09	1.2	6	8	1.2
MC exp. multijet events	0	0	0	0	0	0
Charge flip events (estimated from data)	0	0	0	0	0	94
Fake lepton events (estimated from data)	39	13	120	270	80	51

Various tests of the fit are carried out in order to ensure its stability. As a test of the multijet background constraint and the validity of fitting the $M_{R}'$ distributions in those control regions, the control region fit is instead performed in the number of jets with *p*_T_>30 GeV. The *p*_T_ cut is raised from the baseline selection to make the fit less sensitive to pile-up effects. The expectation for the multijet background in the signal regions is consistent with the main result.

**Fig. 7:**
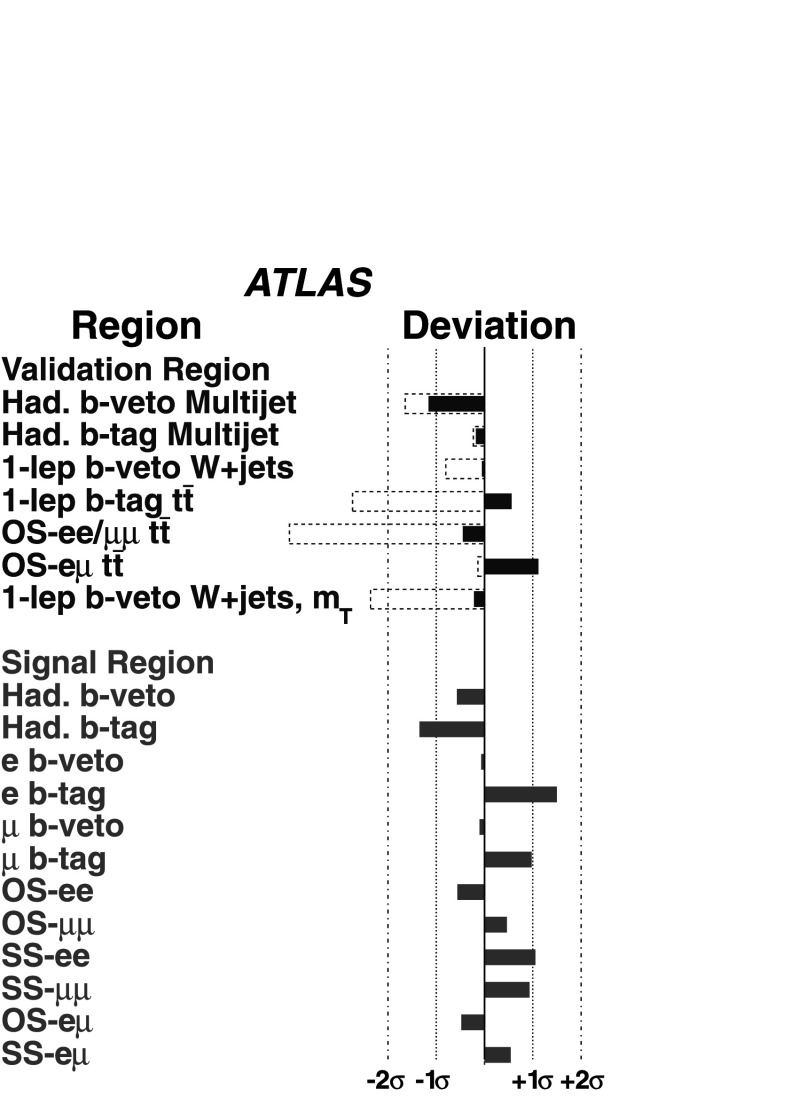
Pull distributions of the numbers of events in the validation regions (VR) and signal regions (SR). The *filled* (*dashed*) bars show the agreement after (before) the background-only fit to the control regions has been performed

The yields and distributions in the validation regions show good agreement with the Standard Model expectation. The significance of the deviation of the observation from the expectation in each of the signal and validation regions are shown in Fig. 7. There is some tension in the pre-fit results between the same-flavour and opposite-flavour dilepton $t\bar{t}$ validation regions, but there is no indication of a systematic mis-modelling of any of the major backgrounds. The yields of all validation regions are within 1.2*σ* of the SM expectations.

The numbers of expected events in each signal region before and after the fit to the control regions are shown in Tables 5 and 6. Additionally, the probability (*p*_0_-value) that a background-only pseudo-experiment is more signal-like than observed is given for each individual signal region. To obtain these *p*_0_-values, the fit in the signal region proceeds in the same way as the control-region-only fit, except that the number of events observed in the signal region is included as an input to the fit. Then, an additional parameter for the non-Standard-Model signal strength, constrained to be non-negative, is fitted. The shape of the distributions in the signal region is neglected in this fit. Therefore, in order to provide tighter constraints on non-Standard-Model production, in some of the high-count signal regions the $M_{R}'$ requirements are tightened. In all other ways, these signal regions follow the definitions in Table 2. Within the fiducial region defined using the same requirements on lepton and jet multiplicities and the razor variables, but using the MC event generator output to define all objects, the typical efficiencies for the models studied are near 100 %. The observed number of events in each of these regions is then compared to the expectation from the Standard Model backgrounds. The significance of the excess is given, along with the model-independent upper limit on the number of events and cross-section times acceptance times efficiency from non-Standard-Model production.

**Table 5:** The number of observed events and the results of the background-only fit to the control regions in the zero- and one-lepton signal regions, for an integrated luminosity of 4.7 fb^−1^. Nominal MC expectations (normalised to MC cross-sections) are given for comparison. The errors shown are the statistical plus systematic uncertainties. The *p*_0_-values and significances are given for single-bin signal regions with somewhat tighter $M_{R}'$ cuts, along with the 95 % Confidence Level upper limit on the number events, *N*_BSM_, and cross-section, *σ*, for non-Standard-Model production within each signal region. In parentheses are given the expected upper limit and the upper limit under a one-*σ* upward (↑) or downward (↓) fluctuation in the observation

Signal region	Had. *b*-veto	Had. *b*-tag	*e* *b*-veto	*e* *b*-tag	*μ* *b*-veto	*μ* *b*-tag
Observed events	4	30	6	13	9	4
Fitted background events	5.5±1.5	39±7	10±2	6.6±1.7	5.5±1.7	4.4±1.3
	Fitted background decomposition					
Fitted top events	0.40±0.14	21±3	2.7±0.9	5.0±1.3	1.7±0.6	3.7±1.1
Fitted *W*/*Z* events	4.9±1.3	3.8±0.7	7.2±1.7	1.2±0.5	3.8±1.3	0.6±0.5
Fitted *WW* diboson events	0.03±0.02	0.029±0.010	0.01±0.02	0.000±0.009	0.001±0.008	0.010±0.005
Fitted multijet events	0.25±0.10	14±5	0±0	0±0	0±0	0±0
Fitted charge flip events	0±0	0±0	0±0	0±0	0±0	0±0
Fitted fake lepton events	0±0	0±0	0.3±0.3	0.5±0.6	0±0	0±0
Expected background events	6.7	55	14	8.5	9.5	5.1
	Expected background decomposition					
MC exp. top events	0.88	30	5.7	6.3	3.4	4.6
MC exp. *W*/*Z* events	5.6	4.0	8.5	1.8	6.1	0.5
MC exp. *WW* diboson events	0.04	0.046	0.01	0.000	0.012	0.010
MC exp. multijet events	0.20	21	0	0	0	0
Charge flip events (estimated from data)	0	0	0	0	0	0
Fake lepton events (estimated from data)	0	0	0.3	0.5	0	0
Tight $M_{R}'$ cut (GeV)	600	1100	600	1100	600	1100
Observed events	4	5	5	6	2	4
Background events	6.2±1.8	13±3	5.3±1.6	2.4±1.0	2.4±1.0	1.9±0.8
*p*_0_-value (Gauss. *σ*)	0.72 (−0.57)	0.91 (−1.35)	0.53 (−0.07)	0.07 (1.50)	0.54 (−0.10)	0.16 (0.98)
Upper limit on *N*_BSM_	5.2 ($6.3^{\uparrow9.4}_{\downarrow 4.3}$)	6.5 ($9.3^{\uparrow12.9}_{\downarrow6.9}$)	6.3 ($6.4^{\uparrow9.5}_{\downarrow4.4}$)	9.0 ($5.5^{\uparrow 8.4}_{\downarrow3.7}$)	4.4 ($4.5^{\uparrow7.1}_{\downarrow3.0}$)	6.8 ($4.8^{\uparrow7.5}_{\downarrow3.2}$)
Upper limit on *σ* (fb)	1.1 ($1.3^{\uparrow2.0}_{\downarrow 0.9}$)	1.4 ($2.0^{\uparrow2.7}_{\downarrow1.5}$)	1.3 ($1.4^{\uparrow2.0}_{\downarrow0.9}$)	1.9 ($1.2^{\uparrow 1.8}_{\downarrow0.8}$)	0.9 ($1.0^{\uparrow1.5}_{\downarrow0.6}$)	1.4 ($1.0^{\uparrow1.6}_{\downarrow0.7}$)

**Table 6:** The number of observed events and the results of the background-only fit to the control regions in the two-lepton signal regions, for an integrated luminosity of 4.7 fb^−1^. Nominal MC expectations (normalised to MC cross-sections) are given for comparison. The errors shown are the statistical plus systematic uncertainties. The *p*_0_-values and significances are given for single-bin signal regions, along with the 95 % Confidence Level upper limit on the number events, *N*_BSM_, and cross-section, *σ*, for non-Standard-Model production within each signal region. In parentheses are given the expected upper limit and the upper limit under a one-*σ* upward (↑) or downward (↓) fluctuation in the observation

Signal region	OS-*ee*	OS-*μμ*	SS-*ee*	SS-*μμ*	OS-*eμ*	SS-*eμ*
Observed events	10	15	11	8	18	18
Fitted background events	12±2	13±2	6±4	4±3	20.±3	14±8
	Fitted background decomposition					
Fitted top events	10.2±1.5	10.7±1.6	0.12±0.04	0.39±0.17	19±2	0.7±0.2
Fitted *W*/*Z* events	0.54±0.10	0.6±0.2	0.16±0.04	0.10±0.04	0.26±0.04	0.33±0.07
Fitted *WW* diboson events	0.4±0.4	0.4±0.5	0.6±0.3	0.6±0.5	0.5±1.0	1.2±0.7
Fitted multijet events	0±0	0±0	0±0	0±0	0±0	0±0
Fitted charge flip events	0±0	0±0	1.6±0.4	0±0	0±0	1.1±0.2
Fitted fake lepton events	1.2±1.3	1.3±1.1	3±4	3±3	0.6±0.6	10.±8
Expected background events	15	16	6	5	24	14
	Expected background decomposition					
MC exp. top events	13.1	14.7	0.13	0.49	23	0.6
MC exp. *W*/*Z* events	0.67	0.4	0.19	0.21	0.27	0.36
MC exp. *WW* diboson events	0.4	0.4	0.7	0.7	0.5	1.2
MC exp. multijet events	0	0	0	0	0	0
Charge flip events (estimated from data)	0	0	1.6	0	0	1.0
Fake lepton events (estimated from data)	1.2	1.3	3	3	0.6	11
*p*_0_-value (Gauss. *σ*)	0.71 (−0.56)	0.32 (0.46)	0.15 (1.05)	0.18 (0.93)	0.68 (−0.48)	0.29 (0.54)
Upper limit on *N*_BSM_	7.3 ($8.8^{\uparrow 12.9}_{\downarrow 6.2}$)	11.1 ($9.4^{\uparrow13.7}_{\downarrow6.6}$)	14.0 ($10.2^{\uparrow14.4}_{\downarrow7.4}$)	11.4 ($8.0^{\uparrow 11.4}_{\downarrow5.7}$)	9.4 ($11.1^{\uparrow16.0}_{\downarrow 7.8}$)	17.7 ($14.9^{\uparrow20.8}_{\downarrow10.8}$)
Upper limit on *σ* (fb)	1.6 ($1.9^{\uparrow2.7}_{\downarrow 1.3}$)	2.4 ($2.0^{\uparrow2.9}_{\downarrow1.4}$)	3.0 ($2.2^{\uparrow3.1}_{\downarrow1.6}$)	2.4 ($1.7^{\uparrow 2.4}_{\downarrow1.2}$)	2.0 ($2.4^{\uparrow3.4}_{\downarrow1.7}$)	3.8 ($3.2^{\uparrow4.4}_{\downarrow2.3}$)

**Fig. 8:**
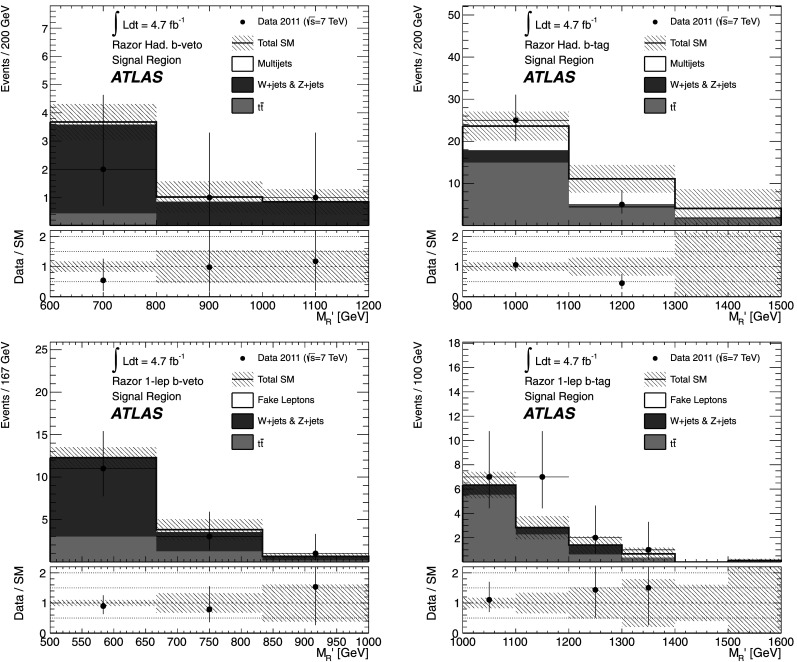
The all-hadronic (*top*) and one-lepton (*bottom*) signal regions with a *b*-tagged jet veto (*left*) and requirement (*right*), after the fit to the control regions has been performed

**Fig. 9:**
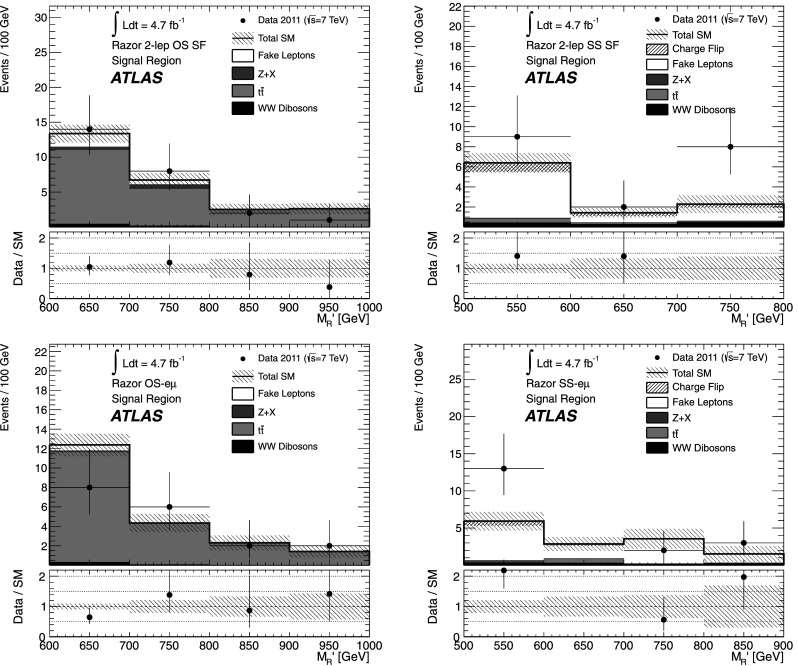
The two-lepton signal regions for same-flavour (*top*) and opposite-flavour (*bottom*) leptons of the same sign (*left*) and opposite sign (*right*), after the fit to the control regions has been performed

The distributions in all signal regions as a function of $M_{R}'$ of background expectations, after the fit to the control region has been performed, are shown in Figs. 8 and 9. No significant deviations from the expected background are found. The most significant excess is 1.50 standard deviations from the expectation, in the one electron, *b*-tagged jet signal region.

## Exclusion results

Using these signal regions, the CL_*S*_ [69] prescription is applied to find 95 % Confidence Level (CL) one-sided limits on the production of SUSY events in various models. The limits on visible cross-section derived in the previous section can be applied to any new physics model. However, in order to compare the exclusion power of the regions to previously published ATLAS results, model-dependent limits are produced. In each case, the exclusion limits are compared to the strongest published ATLAS result. This comparison provides valuable information about the relative strengths of comparable searches using kinematically independent regions. However, as the overlap of the signal regions in this search and the others (discussed in more detail below) is non-zero, a rigorous statistical combination with previously published results is complex and not attempted here.

A likelihood is constructed, taking into account signal shape information provided by the binning of the signal regions. All fitted nuisance parameters, with their correlations, are included in the likelihood. Because the typical $M_{R}'$ of the signal may vary across a signal grid, the use of shape information results in an observed exclusion that is not consistently above or below the expected. The observed limits for the separate zero-, one-, and two-lepton signal regions are also constructed additionally, with all control regions included as constraints.

**Fig. 10:**
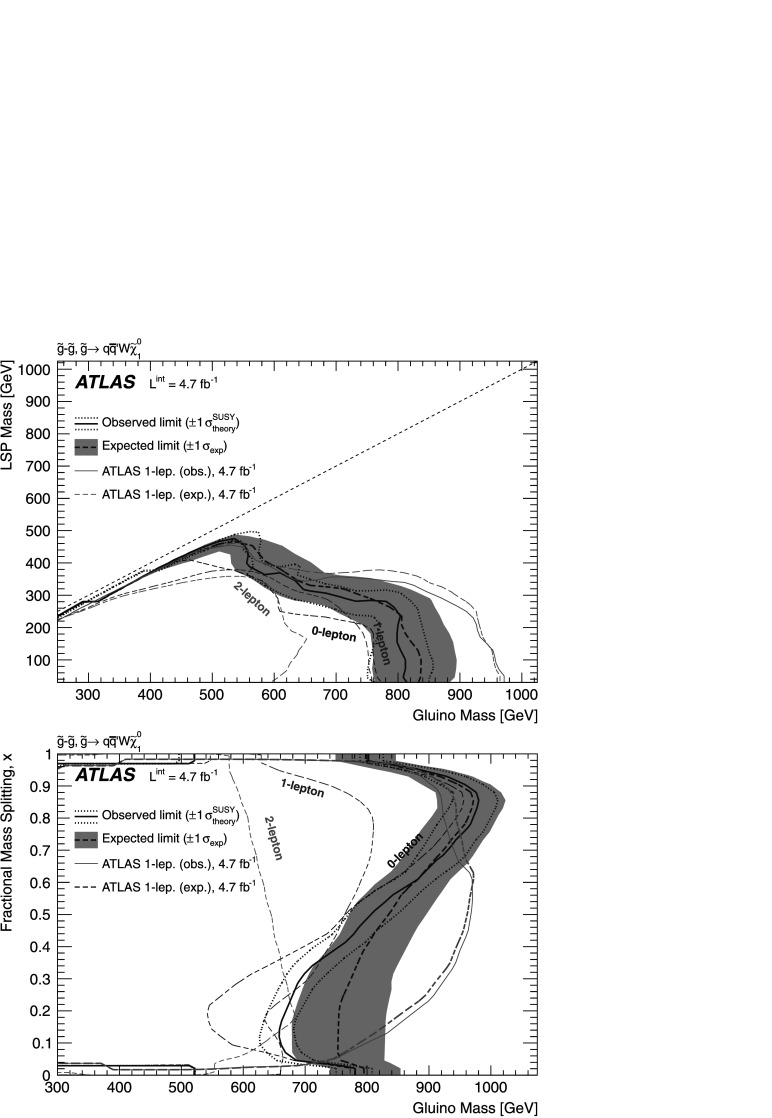
The observed and expected exclusion in a simplified model with gluino pair-production, where the gluinos decay to a chargino via the emission of two quarks and the chargino decays to the LSP and a *W* boson. Top, for the chargino mass exactly half-way between the gluino and LSP mass, in the gluino mass–LSP mass plane, and bottom, for the LSP mass fixed to 60 GeV, in the gluino mass-*x* plane, with *x*=(*m*_chargino_−*m*_LSP_)/(*m*_gluino_−*m*_LSP_). The exclusion is shown for the combination, as well as for each individual channel (labelled 0-lepton, 1-lepton, and 2-lepton). The observed and expected limit of the ATLAS single leptons search [13] (ATLAS 1-lep. (obs.) and ATLAS 1-lep. (exp.), respectively) are indicated as separate contours

Figure 10 shows exclusion contours for a simplified model with gluino pair-production, where the gluinos decay to a chargino and two quarks and the chargino subsequently decays to a *W* boson and the LSP. Two planes are shown for this simplified model. The first fixes the chargino mass to be exactly half-way between the LSP and gluino mass and shows the exclusion in the gluino-mass–LSP-mass plane. The production cross-section falls smoothly and exponentially with *m*_heavy_, while $M_{R}'$ and therefore the acceptance times efficiency for a signal region typically rises with the mass splitting, *m*_heavy_−*m*_LSP_. In the second plane, the LSP mass is fixed to 60 GeV and the exclusion is shown in the gluino mass-*x* plane, where *x*=(*m*_chargino_−*m*_LSP_)/(*m*_gluino_−*m*_LSP_). The zero- and one-lepton signal regions with a *b*-tagged jet requirement do not contribute to the exclusion because these simplified models have only light quarks in the matrix element final state.

At high *x*, although the leptons have high-*p*_T_, the larger branching fraction of the *W* to quarks allows the zero-lepton channel to dominate. At moderate *x*, the leptons allow better discrimination between signal and background in the one- and two-lepton channels. At low *x*, the leptons have too low *p*_T_, and the zero-lepton channel again dominates. At high *x*, the limit set by this analysis exceeds somewhat that of the dedicated 0-lepton and 1-lepton ATLAS searches [10, 13], which have strong $E_{\mathrm{T}}^{\mathrm{miss}} $ requirements and use *M*_eff_ to define signal regions. At low *x* the limit is weaker. This dependence on *x* observed in this analysis, which is not apparent in the other ATLAS searches, is produced by differences in kinematics in these two regions of the plane. At high *x*, the charginos are almost at rest in the lab frame, and the event topology is dominated by a two-body decay, $\tilde{\chi}_{1}^{\pm}\rightarrow W^{\pm}\tilde {\chi}_{1}^{0}$. At low *x*, on the other hand, the chargino is highly boosted, and the topology is dominated by a three-body decay, $\tilde{g}\rightarrow q\bar{q}' \tilde {\chi}_{1}^{\pm}$. Thus, the high-*x* events typically have a higher *R* than the low-*x* events, and the two have approximately the same $M_{R}'$ distribution.

**Fig. 11:**
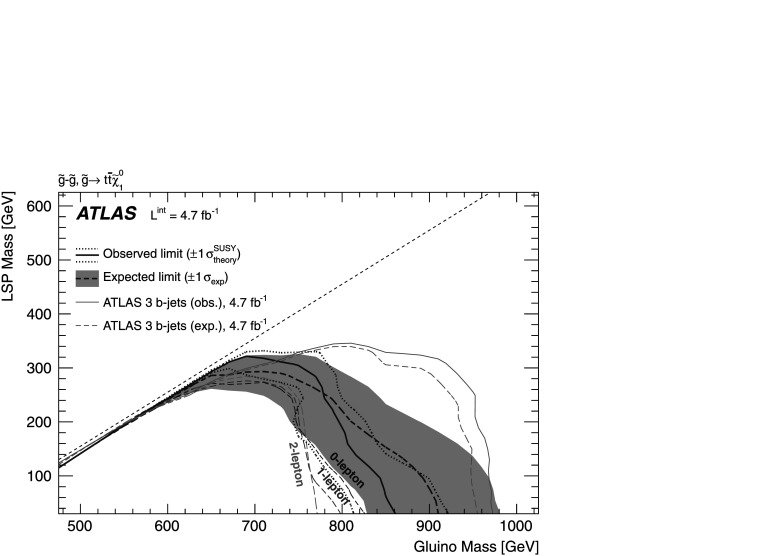
The observed and expected exclusion in a simplified model with gluino pair-production, where the gluinos decay to the LSP via the emission of a $t\bar{t}$ pair. The exclusion is shown for the combination, as well as for each individual channel (labelled 0-lepton, 1-lepton, and 2-lepton). The observed and expected limit of the ATLAS 3 b-jets search [12] (ATLAS 3 b-jets (obs.) and ATLAS 3 b-jets (exp.), respectively) are indicated as separate contours

Figure 11 shows exclusion contours in simplified models with gluino pair-production, where the gluinos decay to the LSP via the emission of a $t\bar{t}$ pair. The exclusion is presented in the gluino mass–LSP mass plane, and, since all top quarks are required to be on-shell, only points with *m*_gluino_>*m*_LSP_+2×*m*_top_ are considered. The zero- and one-lepton signal regions with a *b*-tagged jet veto do not contribute to the exclusion, because these models include four top quarks per event. At small mass splitting, the limits here are somewhat stronger than the ATLAS dedicated multi-*b*-jet analysis [12]. At larger mass splittings, the three *b*-tagged jet requirement suppresses the background substantially while preserving the signal acceptance because of the four tops in the event. The combined limit on LSP mass falls more quickly than that of the multi-*b*-jet analysis because $M_{R}'$ is proportional to the mass splitting in the event, here *m*_gluino_−*m*_LSP_. The zero-lepton razor analysis is limited in this case by the use of the *H*_T_ trigger, which was chosen to avoid a bias in the $M_{R}'$ distribution.

**Fig. 12:**
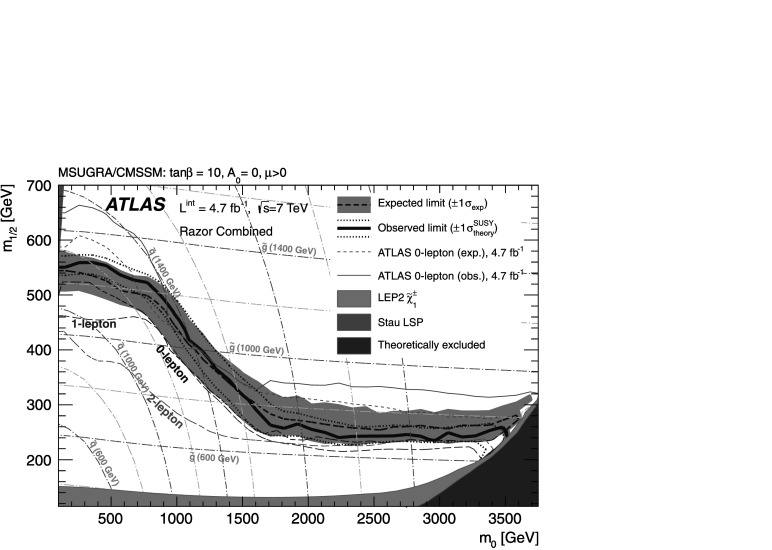
The observed and expected exclusion in a plane of the constrained minimal supersymmetric model. The exclusion is shown for the combination, as well as for each individual channel (labelled 0-lepton, 1-lepton, and 2-lepton). The observed and expected limit of the ATLAS 0-lepton search [10] (ATLAS 0-lepton (obs.) and ATLAS 3 0-lepton (exp.), respectively) are indicated as separate contours

Finally, Fig. 12 shows exclusion contours in a plane of MSUGRA with tan(*β*)=10, *A*_0_=0, and *μ*>0. At low *m*_0_, where squark pair-production is dominant, the zero-lepton channel dominates the exclusion, although it is affected somewhat by the jet multiplicity requirement that is not applied in the dedicated signal region of Ref. [10], which therefore has more stringent limits. The leptonic channels enter at high *m*_0_, particularly where longer decay chains are common. The robustness of the individual limits have also been cross checked by removing some of the control regions. For example, removing the zero- and one-lepton control regions from the calculation of the two-lepton limit, the MSUGRA limit changes by less than 20 GeV in *m*_1/2_. In the *m*_gluino_≈*m*_squark_ region, these limits are consistent with those of earlier ATLAS analyses [10, 11, 13], which rely on transverse information only. In this region, the single mass-splitting scale of the main strong production modes should produce a somewhat sharper peak in $M_{R}'$, allowing an improved limit in the shape fit. At large *m*_0_, the high $M_{R}'$ requirement of the all-hadronic signal regions, resulting from the *H*_T_ trigger use, produce a somewhat weaker limit than the ATLAS multijet analysis [11].

The complementarity of a search using razor variables can be quantified by studying the overlap of the signal regions with the dedicated searches. Various signal models have been studied to understand this overlap, including both simplified models and full SUSY production models. The overlap between the signal regions presented here and other searches in ATLAS [10, 11, 13, 70] is typically 10–50 %, with similar overlaps in the data. The signal regions of this search access kinematic regions that are different from those of the standard searches. In simplified models in particular, the overlap between the dominant signal regions in the standard ATLAS analyses and the signal regions presented here is below 10–15 %. Thus, the regions of SUSY parameter space and kinematic phase space excluded by this search complement those excluded by earlier ATLAS searches using the same data sample.

In the control regions, the overlaps between this analysis and the others are much larger, as they all attempt to select dominant backgrounds with reasonable statistics. The fits that are performed in the various searches, however, look at different properties of the control regions to understand the agreement between data and MC simulation, and therefore the post-fit results may differ somewhat. The background treatments in this search and those previously published are similar enough to consider them correlated in control regions. However, the edges of kinematic phase space explored by the signal regions in these searches may suffer from different features or mis-modelings in MC event generators. Moreover, the treatment of systematic uncertainties and backgrounds varies somewhat between analyses, and because in a simultaneous fit the effects of these uncertainties are convolved, a combination of the various analyses discussed here is beyond the scope of this paper.

## Summary

A search for supersymmetry including final states with zero, one, and two leptons, with and without *b*-tagged jets, in 4.7 fb^−1^ of $\sqrt{s}=7\mbox{ TeV}$ *pp* collisions has been presented. Mutually exclusive signal regions exploiting these final states are combined with the use of variables that include both transverse and longitudinal event information. No significant excess of events beyond the Standard Model background expectation was observed in any signal region. Fiducial cross section upper limits on the production of new physics beyond the Standard Model are shown. Exclusion contours at 95 % CL are provided in SUSY-inspired simplified models and in the constrained minimal supersymmetric extension of the Standard Model.

## Footnotes

1 ATLAS uses a right-handed coordinate system with its origin at the nominal interaction point (IP) in the centre of the detector and the *z*-axis along the beam pipe. The *x*-axis points from the IP to the centre of the LHC ring, and the *y*-axis points upward. Cylindrical coordinates (*r*,*ϕ*) are used in the transverse plane, *ϕ* being the azimuthal angle around the beam pipe. The pseudorapidity is defined in terms of the polar angle *θ* as *η*=−lntan(*θ*/2).
